# Overdispersed gene expression in schizophrenia

**DOI:** 10.1038/s41537-020-0097-5

**Published:** 2020-04-03

**Authors:** Guangzao Huang, Daniel Osorio, Jinting Guan, Guoli Ji, James J. Cai

**Affiliations:** 1 Department of Automation, Xiamen University, Xiamen, 361005 China; 2 National Institute for Data Science in Health and Medicine, Xiamen University, Xiamen, 361005 China; 3 Department of Veterinary Integrative Biosciences, Texas A&M University, College Station, TX 77843 USA; 4 Innovation Center for Cell Signaling Network, Xiamen University, Xiamen, 361005 China; 5 Department of Electrical and Computer Engineering, Texas A&M University, College Station, TX 77843 USA; 6 Interdisciplinary Program of Genetics, Texas A&M University, College Station, TX 77843 USA; 7 Present Address: College of Electrical and Electronic Engineering, Wenzhou University, Wenzhou, 325035 China

**Keywords:** Genetics of the nervous system, Schizophrenia

## Abstract

Schizophrenia (SCZ) is a severe, highly heterogeneous psychiatric disorder with varied clinical presentations. The polygenic genetic architecture of SCZ makes identification of causal variants a daunting task. Gene expression analyses hold the promise of revealing connections between dysregulated transcription and underlying variants in SCZ. However, the most commonly used differential expression analysis often assumes grouped samples are from homogeneous populations and thus cannot be used to detect expression variance differences between samples. Here, we applied the test for equality of variances to normalized expression data, generated by the CommonMind Consortium (CMC), from brains of 212 SCZ and 214 unaffected control (CTL) samples. We identified 87 genes, including *VEGFA* (vascular endothelial growth factor) and *BDNF* (brain-derived neurotrophic factor), that showed a significantly higher expression variance among SCZ samples than CTL samples. In contrast, only one gene showed the opposite pattern. To extend our analysis to gene sets, we proposed a Mahalanobis distance-based test for multivariate homogeneity of group dispersions, with which we identified 110 gene sets with a significantly higher expression variability in SCZ, including sets of genes encoding phosphatidylinositol 3-kinase (PI3K) complex and several others involved in cerebellar cortex morphogenesis, neuromuscular junction development, and cerebellar Purkinje cell layer development. Taken together, our results suggest that SCZ brains are characterized by overdispersed gene expression—overall gene expression variability among SCZ samples is significantly higher than that among CTL samples. Our study showcases the application of variability-centric analyses in SCZ research.

## Introduction

Schizophrenia (SCZ)—one of the most severe psychiatric disorders—affects about 1% of the general population^1–3^. The disorder manifests itself in many different forms and includes both positive behaviors, such as delusions, hallucinations, and disorganized speech, and negative behaviors, such as the absence of reaction, loss of interest in everyday activities, and lack of feeling or emotion. SCZ affects patients differently. People with the disorder vary widely in their symptoms, course of illness, and treatment response^4^. A reliable clinical typology of SCZ has proved difficult to develop^5–7^. Assessment of clinical outcomes in SCZ is challenging^8,9^. Patients diagnosed with SCZ can be classified into those with and without neurodevelopmental impairment^10–12^. The former category is likely to be due to the impact of risk alleles, copy number variants (CNVs), or early environmental insults such as hypoxic damage to the hippocampus. The latter is more likely to be due to affective dysregulation. Detailed neuroimaging and many measures of psychological variables can also be used to classify SCZ patients into distinct subgroups^13–16^. The existence of multiple ways to classify SCZ underscores the marked between-patient variability and within-category heterogeneity associated with the disorder. Thus, SCZ is a highly heterogeneous group of disorders rather than a single disease^17–19^.

Although SCZ was described more than 100 years ago, the exact etiology and genetic mechanism of SCZ are still unclear. With an upper bound estimate of heritability of 80%^20^, the risk of SCZ is clearly under the substantial genetic influence. Numerous common single nucleotide polymorphisms (SNPs) (refs ^21–23^) and CNVs (refs ^24–27^) have been identified to be associated with the SCZ risk. Nevertheless, like many other complex diseases, SCZ has a polygenic architecture^21,22,28^, and is influenced by environmental factors^16^, making it difficult to pinpoint causal mutations.

Gene expression data of SCZ patients has been collected and integrated into analyses^29–33^, in order to improve mechanistic interpretations of risk alleles, or directly identify dysregulated genes in relevant tissues. Most studies of SCZ transcriptome adopt the method of differential expression (DE) aimed at the identification of genes expressed significantly differently in SCZ patients than unaffected controls (CTL). However, given the fact that SCZ is highly heterogeneous, we argue that the DE analysis may not be sufficient as it treats SCZ patients as a homogeneous group of individuals, assuming gene expression in all SCZ samples, compared with that in all CTL samples, is consistently up- or downregulated. Indeed, a robust overlap between sets of DE genes identified in different SCZ transcriptome studies has been rarely observed. Many of statistically significant DE genes cannot be individually connected with any of the current pathophysiological hypotheses of the disease either.

Here, we consider a complementary alternative. We assume disease-relevant genes are expressed more variably in SCZ patients than CTL individuals and therefore lead to an ‘overdispersion’ in gene expression in the SCZ group. The rationale behind our argument is that patients affected with SCZ are a heterogeneous group of individuals: it is not simply that they share few or no symptoms in common^34^; rather, etiologically, every patient with SCZ is ‘ill’ in his or her own way. This can be summarized into the ‘Anna Karenina principle’ for SCZ, in which gene expression of affected individuals varies more than that of healthy individuals, matching with Leo Tolstoy’s dictum that ‘happy families are all alike; each unhappy family is unhappy in its own way’. We set out to test the hypothesis that overdispersed gene expression in SCZ is a common and important consequence of transcriptional dysregulation. The phenomenon should be more pronounced for genes and pathways that underlie SCZ pathogenesis. The pattern is easily missed or discarded by common workflows such as those implemented in the DE analysis.

The structure of this paper is as follows. First, we represent the results of our differential variability (DV) analysis on single genes, showing an overwhelming pattern of increased variability at the single-gene level associated with SCZ. Second, we introduce a newly developed, multivariate DV analysis method. Through the application of this method, we show that multiple gene sets, whose enriched functions are SCZ related, have higher expression variability among the SCZ subjects. Third, we show the contribution of common genetic variants to expression variability. To the best of our knowledge, this kind of variability-centric analysis has not been done with data sets from the SCZ cohort. We conclude by providing the interpretation of our results in the context of gene discovery and implications in the personalized intervention of SCZ.

## Results

### Single genes with higher expression variability among SCZ samples

We obtained the normalized gene expression data from the CommonMind Consortium (CMC) study^29^. The data were generated using RNA sequencing (RNA-seq) from the dorsolateral prefrontal cortex of individuals affected with SCZ and unaffected control (CTL) individuals. Our analyses were done with data from 212 SCZ and 214 CTL individuals, all with European ancestry. For each gene, we used the Brown–Forsythe (B–F) test (ref. ^35^) to determine whether there is a significant difference in group variances between SCZ and CTL. At the level of 5% false discovery rate (FDR), we identified 88 differentially variable (DV) genes (Supplementary Table 1), including 87 with a greater expression variance in SCZ than CTL and one (*TAMM41*) with a smaller expression variance in SCZ than CTL. Thus, at the single-gene level, greater between-individual expression variability is an overwhelming feature of SCZ. Illustrative examples, showing DV genes’ expression levels across CTL and SCZ samples, are given in Supplementary Fig. 1. The overdispersed expression in SCZ is robust against outlying samples—i.e., the pattern was not driven by outlying individuals with extreme expression values in a large number of genes. Using one of DV genes, *ZBTB24*, as an example, we intentionally removed two SCZ samples with the highest and lowest expression value, respectively. Our purpose was to assess the impact of these two outliers on the significance of the statistical test for the DV gene detection. The removal of these two extreme samples had no qualitative influence on the outcome of the B–F test (Supplementary Fig. 2). These results suggest that increased gene expression variability in SCZ is unlikely to be due to the existence of a small number of SCZ samples with extremely high or low expression levels. Instead, it is due to a systematic overdispersion of gene expression among SCZ samples.

Furthermore, using a data set from the BrainSeq project^36^, which contains the patients’ information on the use of medicine, we estimated the contributions of antipsychotics and antidepressants to gene expression variability in SCZ samples. First, we identified DV genes expressed more variably in SCZ than CTL. Then, for each of these DV genes, we compared its expression variance in SCZ patients received antipsychotics treatment with that in SCZ patients not received antipsychotics treatment. No gene was found to be significant using the B–F test with the multiple test correction. Finally, we also compared the distribution of B–F test *p*-values and the distribution obtained using randomly shuffled data. No difference was revealed either (see Supplementary Fig. 3 for QQ plots). We also plotted the expression levels of selected DV genes to visualize their distribution pattern in treated and untreated SCZ patients (Supplementary Fig. 4). Taken together, these analyses suggest that the contribution of the use of antipsychotics and antidepressant to gene expression variability in SCZ seems minimal.

**Fig. 1:**
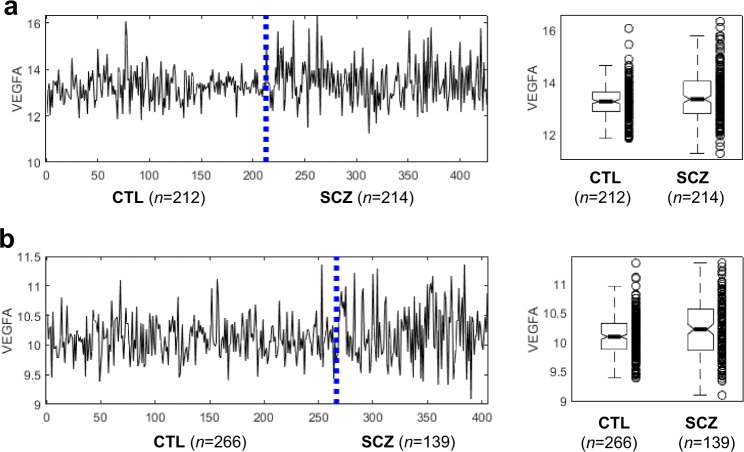
Expression profiles of *VEGFA* show more dispersed expression in SCZ than CTL. **a** Normalized expression values in 212 CTL and 214 SCZ samples generated by the CMC study. B–F test *p*-value = 3.6e−6. **b** Normalized expression values in 266 CTL and 139 SCZ samples, as reported in ref. ^42^. B–F test *P*-value = 1.7e−9.

Using the gene ontology (GO) enrichment test^37^, no significantly enriched function was found to associated with the 88 DV genes, suggesting marked functional diversity of these genes. One of the most salient examples is *VEGFA*, a gene encoding vascular endothelial growth factor (Fig. 1a). This signal protein, VEGFA, stimulates vasculogenesis and angiogenesis^38^, playing an important role in neurogenesis, neuronal differentiation, and neuroprotection and regeneration of central nervous system (CNS) cells^39–41^. Data from an independent microarray-based study confirms the higher *VEGFA* expression variability in SCZ samples (Fig. 1b)^42^. In addition to *VEGFA*, *BDNF* is a member of the neurotrophin family of growth factors related to the canonical nerve growth factor; *NECTIN2* encodes a single-pass type I membrane glycoprotein implicated in Alzheimer’s disease; *AIF1* encodes allograft inflammatory factor found in activated macrophages in tissues with inflammation (Supplementary Fig. 1). Furthermore, *HPS5*, *STAR*, *TMEM125*, *RPN2*, and *DNAH1* loci are known to contain genetic variants associated with SCZ^43–47^.

### Gene sets with higher expression variability among SCZ samples

Next, we turned our focus from single genes to gene sets. To extend our analysis to gene sets, we adapted the procedure for analyzing multivariate homogeneity of group dispersions^48^. This procedure is a multivariate analog of Levene’s test for homogeneity of variances and has been widely used in ecology, for example, in defining the variability in species composition. In the proposed method, we used Mahalanobis distance (MD) to replace Euclidian distance originally used in the procedure. This adaptation is essential to account for collinearity in expression levels of genes in each gene set across samples^49,50^. To increase the power of the test, we introduced further modification in our MD-based test (see Methods for details). We applied this modified version to gene sets, which are pre-defined using GO terms in ontologies of biological process and molecular function. We identified 110 gene sets with greater multivariate expression variance in SCZ, all with a nominal *p*-value < 0.05 (Supplementary Table 2).

The top 30 most significant gene sets are given in Table 1. The most significant gene set contains 15 out of 20 genes involved in forming the *phosphatidylinositol 3-kinase (PI3K) complex*. The PI3K signaling pathway is known to be implicated in SCZ^51–54^. Among the rest of gene sets, many are related to brain development such as (1) *cerebellar cortex morphogenesis*, (2) *cerebellar cortex formation*, (3) *neuromuscular junction development*, (4) *cerebellar Purkinje cell layer development*, (5) *cell differentiation in hindbrain*, and (6) *regulation of astrocyte differentiation*, suggesting that SCZ brains are characterized by abnormally high expression variance in genes involved in the brain development. This finding is consistent with the general consensus that SCZ is a brain disorder with structural and functional changes in the cortex and the connections between different cortical regions^11,16,55^. In addition to these gene sets pertaining to brain development, several other gene sets are likely to be implicated in SCZ. These include *necrotic cell death*^56,57^, *regulation of endoplasmic reticulum unfolded protein response*^58^, and *folic acid-containing compound metabolic process*^59–61^. For each significant gene set, we use the method of Garthwaite–Koch partition to estimate the relative contribution of each gene in the gene set to MD^62^. We present the top five genes that contribute most to the MD for each gene set in Table 1.

**Table 1:** Functional gene sets showing higher expression variability in SCZ compared with CTL.

	Gene set	Number of genes^a^	*P*-value	Top 5 genes contributing most to multivariate expression variability of the gene set
1	GO_PHOSPHATIDYLINOSITOL_3_KINASE_COMPLEX	15 (20)	7.47E−05	PIK3R1, PIK3R4, NRBF2, PIK3CA, PIK3C3
2	GO_REGULATION_OF_B_CELL_PROLIFERATION	28 (55)	1.28E−04	AHR, INPP5D, PKN1, SLC39A10, CD74
3^b^	GO_CEREBELLAR_CORTEX_MORPHOGENESIS	23 (30)	1.85E−04	KNDC1, SPTBN2, LDB1, SERPINE2, FAIM2
4	GO_REGULATION_OF_ENDOPLASMIC_RETICULUM_UNFOLDED_PROTEIN_RESPONSE	22 (28)	2.48E−04	PIK3R1, DAB2IP, HSPA5, BBC3, POMT2
5	GO_GENETIC_IMPRINTING	15 (20)	4.42E−04	ARID4A, BRCA1, ARID4B, ZFP57, MECP2
6	GO_POSITIVE_REGULATION_OF_STRIATED_MUSCLE_CELL_DIFFERENTIATION	24 (52)	5.44E−04	HOPX, CYP26B1, MAPK14, PROX1, THRA
7	GO_ENDORIBONUCLEASE_ACTIVITY	33 (54)	5.57E−04	FEN1, POP4, DROSHA, RNASEH2B, RPP30
8	GO_INSULIN_LIKE_GROWTH_FACTOR_RECEPTOR_SIGNALING_PATHWAY	14 (14)	7.87E−04	PIK3R1, TSC2, GRB10, AKT1, IGF2R
9	GO_LIPOPROTEIN_PARTICLE_RECEPTOR_ACTIVITY	12 (16)	9.76E−04	LDLR, LRP6, OLR1, SCARB1, LRP10
10	GO_RNA_POLYMERASE_II_ACTIVATING_TRANSCRIPTION_FACTOR_BINDING	24 (36)	9.83E−04	LDB1, BHLHE40, BEX1, RB1, NCOR1
11	GO_FOLIC_ACID_CONTAINING_COMPOUND_METABOLIC_PROCESS	22 (29)	1.10E−03	MTHFD1, MTRR, ALDH1L1, SLC19A1, FTCD
12^b^	GO_CEREBELLAR_CORTEX_FORMATION	19 (22)	1.74E−03	KNDC1, LDB1, CDK5, FAIM2, CEND1
13	GO_NEGATIVE_REGULATION_OF_VASCULATURE_DEVELOPMENT	40 (80)	1.79E−03	ITGB1BP1, PDCD10, SEMA4A, PML, DAB2IP
14	GO_RETROGRADE_TRANSPORT_VESICLE_RECYCLING_WITHIN_GOLGI	19 (24)	1.97E−03	OPTN, RAB6A, COG1, PACS1, GOLGA1
15^b^	GO_NEUROMUSCULAR_JUNCTION_DEVELOPMENT	28 (36)	2.13E−03	UNC13A, CACNB1, ETV5, AFG3L2, ERBB2
16^b^	GO_CEREBELLAR_PURKINJE_CELL_LAYER_DEVELOPMENT	20 (24)	2.39E−03	LDB1, SPTBN2, SEZ6L, UQCRQ, ATP2B2
17	GO_NEGATIVE_REGULATION_OF_EPITHELIAL_CELL_APOPTOTIC_PROCESS	18 (35)	3.55E−03	SEMA5A, SCG2, WFS1, TEK, KRIT1
18	GO_PYRIMIDINE_DEOXYRIBONUCLEOTIDE_METABOLIC_PROCESS	14 (18)	4.02E−03	NT5C, MBD4, NEIL2, DTYMK, UNG
19	GO_NEGATIVE_REGULATION_OF_CIRCADIAN_RHYTHM	11 (17)	4.14E−03	ADORA1, CRY2, PER2, SUV39H2, SIN3A
20	GO_NEGATIVE_REGULATION_OF_OXIDOREDUCTASE_ACTIVITY	13 (26)	4.29E−03	NFKB1, ATP2B4, TMLHE, CNR1, PRDX5
21	GO_ACTIVATING_TRANSCRIPTION_FACTOR_BINDING	40 (57)	4.51E−03	LDB1, BHLHE40, RB1, BEX1, NCOR1
22	GO_FOUR_WAY_JUNCTION_DNA_BINDING	13 (15)	4.97E−03	HMGB3, MECP2, HMGB1, RAD51C, MSH6
23^b^	GO_CELL_DIFFERENTIATION_IN_HINDBRAIN	15 (21)	5.24E−03	KNDC1, LDB1, FAIM2, CEND1, CACNA1A
24^b^	GO_CEREBELLAR_PURKINJE_CELL_LAYER_MORPHOGENESIS	11 (14)	5.59E−03	LDB1, SPTBN2, FAIM2, CACNA1A, ATP2B2
25	GO_ESTABLISHMENT_OF_MITOTIC_SPINDLE_ORIENTATION	16 (20)	6.20E−03	HTT, SPRY1, SPRY2, PAFAH1B1, NUMA1
26	GO_PTERIDINE_CONTAINING_COMPOUND_METABOLIC_PROCESS	28 (36)	6.64E−03	PTS, MTR, MTHFD1, MTRR, ALDH1L1
27^b^	GO_HINDBRAIN_MORPHOGENESIS	29 (40)	7.09E−03	KNDC1, SPTBN2, LRP6, FAIM2, LDB1
28^b^	GO_REGULATION_OF_ASTROCYTE_DIFFERENTIATION	19 (27)	7.42E−03	BMP2, PRPF19, SERPINE2, NOTCH1, NF1
29	GO_PHOSPHATIDYLCHOLINE_BIOSYNTHETIC_PROCESS	18 (27)	8.39E−03	ACHE, FGF7, LPIN1, SLC44A5, PCYT1A
30	GO_REGULATION_OF_GENE_EXPRESSION_BY_GENETIC_IMPRINTING	12 (16)	8.53E−03	ARID4A, BRCA1, ARID4B, ZFP57, DIRAS3

For each gene sets, the top five genes that contribute most to expression variability are given.

^a^Number of genes used in the analysis (number of genes in the gene set).

^b^Gene sets with functions involved in the central and peripheral nervous system.

**Fig. 2:**
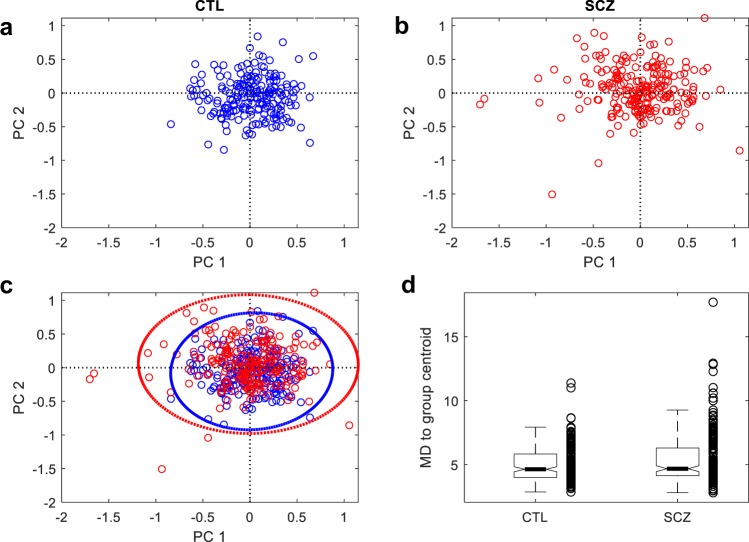
Gene set, *cerebellar cortex morphogenesis*, show more dispersed expression in SCZ. The PCA analysis was performed with the gene set expression matrix of pooled samples that contain all SCZ and CTL samples. **a** Distribution of CTL samples on the PCA space defined by the first two PCs. SCZ samples are made invisible by plotting in white color. **b** Distribution of SCZ samples with CTL samples made invisible. **c** Distribution of all samples in the PCA space. Dashed lines indicate the 99% confidence ellipses. **d** Boxplot of MD vectors in SCZ and CTL groups, showing the high within-group variance in SCZ.

To illustrate the overdispersion pattern of gene set expression in SCZ, we used the gene set of *cerebellar cortex morphogenesis* as an example (Fig. 2). We extracted the expression data of 23 genes in the gene set, pooled SCZ with CTL samples, and performed principal component (PC) analysis. On the first and second PC space, SCZ samples are more dispersedly distributed than CTL samples (Fig. 2a–c). Accordingly, the variance in the MD of individual SCZ samples to the centroid is significantly greater than that of CTL samples (Fig. 2d).

We also identified nine gene sets showing the opposite pattern, i.e., smaller expression variance in SCZ than CTL (Supplementary Table 3). These include two related to CNS: (1) neurexin family protein binding and (2) neuron cell-cell adhesion.

### Statistical power analysis

We used computer simulations to conduct a power analysis for our MD-based multivariate test for homogeneity of variances. As mentioned, our test is an extension of the procedure of Anderson^48^. The simulations were done with combinations of a series of sample sizes and varying levels of variance difference between case and control groups (see Methods for details). We considered a balanced design with the sample size of the case group equals to that of the control group. The result of the power analysis shows that the power of our extended test starts to increase when the sample size per group is over 800 (Supplementary Fig. 5a). The test becomes highly sensitive when the sample size per group reaches 1000—in this case, when variances of the two groups differ by twofold, the statistical power of our test can reach 80%. To increase the power of our MD-based test, we proposed a modified version of it (see Methods). With this modification, the power of our test increases substantially (Supplementary Fig. 5b). Our modified MD-based test requires ~700 samples to reach 80% power. Despite this improvement, the power analysis suggests that the sample size we used in our real data analysis (212 and 214 for SCZ and CTL, respectively) was still too small. Additional studies are needed to investigate differences in gene set expression variability between groups further.

### Genetic variants contribute to dispersed gene expression in SCZ

**Fig. 3:**
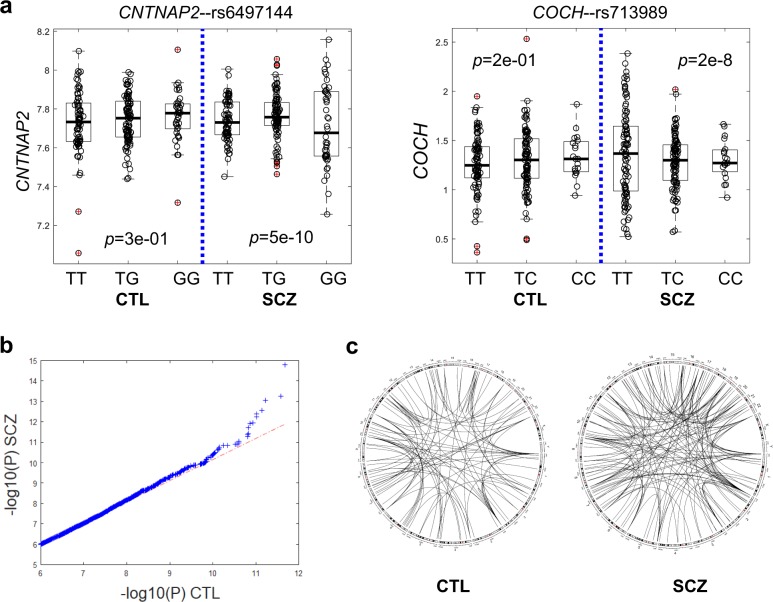
Genetic variants associated with gene expression variability in SCZ. **a** Two examples of SCZ-specific evQTLs, showing significant differences in expression variances between genotypes in SCZ but not in CTL. *P*-values of the B–F test for three genotype groups in SCZ and CTL, respectively, are given. **b** Quantile-quantile plot of *P*-values for evQTL associations in SCZ (*y*-axis) against those in CTL (*x*-axis). **c** Difference in the density of highly significant evQTL associations between variants and genes in SCZ and CTL.

To assess the contribution of genetic variants to the gene expression dispersion in SCZ, we conducted the expression variability QTL (evQTL) mapping analysis^63,64^. An evQTL is an SNP with alleles associated with group variance in gene expression. In this case-control study setting, we were more interested in SNPs with different effects on SCZ and CTL, and thus we set out to identify SCZ-specific evQTLs. We tested all common SNPs segregating in SCZ and CTL, i.e., minor allele frequency (MAF) > 0.15 in both populations, to identify those with genotypes associated with gene expression variance in SCZ (*p* < 1e−7, B–F test) but not in CTL (*p* > 0.05, B–F test, Fig. 3a). We identified 2503 SCZ-specific evQTLs involving 1453 distinct autosomal protein-coding genes (Supplementary Table 4, see Supplementary Fig. 6 for more examples). For comparison, we used the same procedure and *p*-value cutoffs to identify CTL-specific evQTLs with SCZ samples as the background group. We identified 2076 CTL-specific evQTLs involving 1277 genes. Although the number of CTL-specific evQTLs is comparable to that of SCZ-specific evQTLs, a q–q plot shows that the overall statistical significance is much stronger for SCZ-specific evQTLs, especially for those highly significant ones (Fig. 3b). We plotted the links between these highly significant evQTLs (*p* < 1e−9) with their target genes for both SCZ and CTL results. Most of these relationships are *trans*-acting and more SCZ-specific evQTLs connect with target genes and form a denser picture than CTL-specific ones do (Fig. 3c).

## Discussion

Our analysis focuses on the differences in expression variances between groups. The DV approach we adopted here could be rooted in the theory of dynamics of correlation and variance in systems under the load of environmental factors^65^. For heterogonous disorders like SCZ, we show that the DV approach is powerful in identifying significant genes and characterizing biological pathways and processes critical to the disorder. DV approach is complementary to the more commonly used DE method. So far, identified DE genes between SCZ and CTL show tremendous functional diversity and often fail to form supported, functionally interpretable gene sets or pathways (with few exceptions such as the AMPA GluR1 receptor subunits and the glutamatergic neurotransmission pathway)^66^, which is not unexpected under the assumption of substantial heterogeneity in SCZ pathophysiology^32^.

Overdispersion is the term we borrow from statistics to describe the presence of greater variability (statistical dispersion) in gene expression data in SCZ than would be expected based on that in CTL. The overdispersion pattern we observed in SCZ is in line with the fact that clinical heterogeneity amongst people diagnosed with SCZ is high, which has hampered the development of individual treatment and research into new treatment strategies. We assume that key pathways underlying SCZ risk are disrupted via many different causes—genetic, epigenetic, and environmental. The consequence of these disruptions is collectively reflected as dysregulated gene expression, which is, in turn, characterized by an increased level of group dispersions (variances). Thus, the degree of gene expression dispersion is an excellent predictor of functional disruptions. Even if the illness of SCZ in every affected individual arises from a different specific cause, each will nonetheless share disruption of related key biological processes.

Furthermore, the effects of environmental factors may accumulate throughout the progress of chronic SCZ. In such cases, a lifetime of stress, smoking, and antipsychotic drug administration are examples of these environmental factors, which may take effect through interacting with genotypes of individual SCZ patients. Thus, through assessing the gene expression in the brain tissue, we identify the related pathways dysregulated in patients affected by SCZ. Such dysregulated expression is a transcriptional echo of the pathways and mechanisms associated with the onset of the illness.

In this study, *VEGFA*, the gene encoding vascular endothelial growth factor, stands out as the most significant single DV gene, cross-validated with two independent expression data sets^29,42^. The discovery of this significant gene is consistent with accumulating evidence indicating that SCZ is accompanied by abnormal vascularization^38,67,68^. The role of VEGF in causing neurovascular dysfunction has been known to be correlated with hypoxia-ischemia insults during early life and is strongly associated with cognitive dysfunction^38,41^. Clinical studies examining peripheral VEGF levels in SCZ versus CTL have yielded conflicting results. While some studies found elevated serum VEGF concentrations in individuals with SCZ^69,70^, others revealed no significant difference^71–74^, or lower concentrations^75,76^. The extremely high between-individual variability in *VEGFA* expression may explain such conflicting findings.

One of the caveats of our study is that the influence of antipsychotics on gene expression in SCZ patients could not be easily controlled. Antipsychotic drugs are widely used to treat SCZ. In the clinical settings, because antipsychotics have large inter-individual differences in efficacy, there is no methodology to predict the effect of antipsychotics^77^. Although much progress has been made on antipsychotic drug development, precise mechanisms behind the action of antipsychotics are poorly understood^78,79^. Non-genetic factors such as epigenetic modifications are known to be involved in the effect of antipsychotics^80^. For *VEGFA*, it is known that antipsychotics influences plasma VEGF levels, but the direction of the influence varies (refs ^76,81^. cf. ref. ^72^).

An important contribution of our study is to develop the homogeneity test of multivariate dispersions further using an MD-based extension. The initially proposed procedure by Anderson (ref. ^48^) is flexible enough to allow any distance measure to be adopted. We have previously used a slightly different implementation of the test to identify dysregulated gene sets in autism spectrum disorder^82,83^. Other implementations include the one based on the means of within-group distances, which does not require group center calculations to obtain the distance statistic^84^, and the one to compare *k* populations based on Fréchet variance for general metric space valued data objects, with emphasis on comparing means and variances^85^. The most important feature shared by these tests is their capability of capturing the between-group difference in multivariate covariance of variables. Our method, when applied to gene sets, has proved to be useful in identifying functionally meaningful gene sets. Our results reveal that transcriptional dysregulation in genes responsible for brain development is significantly implicated in SCZ, which is in line with the consensus of SCZ being a brain disorder^11,16,55^. Our analysis with gene sets supports that the cell-death pathways activated via extrasynaptic NMDA receptors^86,87^, folic acid^88,89^, and metallopeptidases^90^, are implicated in SCZ.

We also report the opposite pattern detected in several gene sets, for which expression variability is reduced rather than elevated in SCZ. These include genes with the molecular function of interacting selectively and non-covalently with neurexins that act as cell recognition molecules at nerve terminals. Thus, despite its scarcity, such an underdispersion pattern in gene expression variability may also have important implications compared with the overdispersion pattern. The underdispersion pattern may even reflect a more severely dysregulated status of gene expression in SCZ.

We explored the relationship between genetic variants and expression variability. Previously, we adopted the ‘vQTL’ model^91^, and applied the model-based method to human gene expression data^63^. Up to date, a large number of evQTLs have been identified with different human gene expression data sets^64,92,93^. Here, in the present study, we detect evQTLs in SCZ and CTL samples, separately, and highlight differences in the number and statistical significance of evQTLs between SCZ and CTL. We found more statistically significant evQTLs in SCZ than in CTL, suggesting that common genetic variants may play a more important role in shaping the gene expression variability in SCZ. It is not clear though whether these genetic variants exert destabilizing function on their own or act through interacting with each other^92^. Nevertheless, these results have clinical implications. For example, *CALM1*–rs2123259 (GT) and *HTR1A*–rs12440923 (TC) are two SCZ-specific evQTLs identified in this study (Supplementary Table 3). *CALM1* encodes for calmodulin 1 and *HTR1A*, serotonin 1A receptor (or 5-HT_1A_ receptor). Both gene products are targets of antipsychotic drugs. For example, aripiprazole is a partial agonist at the 5-HT_1A_ receptor; chlorpromazine binds to calmodulin to exert an inhibitory effect and exhibited antagonist activity at serotonin 1A receptors^94,95^. According to the evQTL pattern, the expression level of *CALM1* in SCZ patients with TT genotype at rs2123259 (chr15:87257140_hg19) is highly variable from patient to patient, and the same as for the *HTR1A* expression in patients with CC genotype at rs12440923 (chr15:93164993_hg19). This information ought to be taken into account in antipsychotic medication administration.

In summary, we proposed new analytical methods to reveal overdispersed gene expression in SCZ. These methods are based on statistical tests for homogeneity in group variances. We showed the application of our methods in the discovery of genetic underpinnings of SCZ that are notoriously difficult to determine. We identified single genes as well as gene sets showing greater gene expression variability in SCZ patients but not in unaffected controls. Functional interpretation of these genes points us to the dysregulation of brain function pertaining to a number of new mechanisms. The highly variable expression of these genes in SCZ is associated with the heterogeneous nature of this disorder, which is characterized by varying clinic presentations and individualized symptoms. As technologies for high-throughput data generation become more common and affordable, the sample size per study will continually increase, which will greatly improve the power of variance-based statistical inference. Given these, we anticipate our study inspires new conceptual development toward variability-centric analyses in SCZ research.

## Methods

### Ethical statement

This study was exempt from the institutional review board (IRB) review at Texas A&M University; no identifiable private information or identifiable biospecimen was used in the analyses.

### Data sets

The gene-level expression data used for the analyses in this study was generated as part of the CommonMind Consortium (CMC) study^29^. The expression matrix was derived from the raw read count matrix through a series of normalization and adjustment. Briefly, 16,423 genes with at least one CPM (read counts per million total reads) in at least 50% of the individuals were retained and processed. The initial normalization was done using voom^96^. Weighted linear regression was then performed for each gene to control for known covariates. The data was further adjusted for hidden variables detected by surrogate variable analysis (see ref. ^29^ for details). We downloaded the processed expression data matrix from the CMC Knowledge Portal (see Data availability). From this data matrix, we extracted data generated from European ancestry individuals, including 212 SCZ and 214 CTL samples. The SCZ group contains 77 female (35.8%) and 137 male (64.2%) donors; the CTL group that contains 91 female (42.9%) and 121 male (57.1%) donors (see Supplementary Table 5 for more details). We used this European data set throughout all subsequent analyses. In addition to the CMC data, an independent, microarray-based gene expression data, collected by a study of multiple psychiatric disorders^42^, was obtained. From this microarray data set, we extracted expression data for 139 SCZ and 266 CTL samples. We also obtained the data from the Phase1 study (http://eqtl.brainseq.org/phase1) of the BrainSeq project^36^. This data set contains information about the use of antipsychotic medications, allowing the investigation of the influence of medications on gene expression variability among SCZ patients.

### Test for homogeneity of expression variances of single genes

Brown–Forsythe (B–F) test was applied to each gene to test for the significant difference in expression variance between subjects of the SCZ and CTL groups. B–F test is a statistical analysis related to Levene’s test—both are tests for homogeneity of variance. B–F test involves determining an absolute deviation score from group medians, while Levene’s test from the group means. After running the B–F test for all individual genes, we used the Benjamini–Hochberg (B–H) procedure to control the FDR of hypothesis tests^97^.

### MD-based multivariate test for homogeneity of expression variances of gene sets

Anderson (2006) proposed a distance‐based test of homogeneity of multivariate dispersions for a one‐way ANOVA design^48^. To identify gene sets with differential multivariate expression variability between SCZ and CTL, we adopted the ‘Anderson06’ test and applied it to functional gene sets. These functional gene sets are pre-defined in the MSigDB v5.2^98^. We selected those defined with GO ontologies of biological process and molecular function. For a given gene set, we measured multivariate expression dispersion (variance) of genes in SCZ and CTL separately. We calculated Mahalanobis distance (MD) of each SCZ and CTL individuals to their group centroids in the multivariate space. Compared with the Euclidean distance used in the original Anderson06 test, MD is a more appropriate distance metric for gene set expression, because expression levels of different genes in a gene set are likely to be correlated^49,50^. To test if the dispersions (variances) of SCZ and CTL groups are different, MDs of group members to the group centroid were subject to ANOVA^48^. We call our method the MD-based Anderson06 test. To increase the statistical power, we introduced a modified version of the MD-based Anderson06 test. In the modified version, values of MD distance metric obtained from all samples (SCZ and CTL) were pooled, and the top 90% percentile of values were multiplicated with a constant that equals to the maximum of MD values, keeping the rest of MD values unchanged. After this treatment, the rest of the steps such as the ANOVA test remained the same as described above. This modification was found to be able to increase the power of the test markedly (Supplementary Fig. 4). Therefore, the modified version was used throughout our analyses for gene sets. Finally, to determine which genes in a gene set have the most influence on the MD, we used the method proposed by Garthwaite and Koch^62^.

### Power analyses

We conducted simulations to evaluate the power of the MD-based Anderson06 test. We fixed the size of the input gene set at 23, based on the number of genes in the actual gene set of *GO_CEREBELLAR_CORTEX_MORPHOGENESIS* (Table 1). We also fixed the covariance matrices, ∑_0_ and ∑_1_ of the input gene set for CTL and SCZ samples, respectively, using the values calculated from the actual expression data of the gene set. We varied values of two parameters: (1) the sample size per group, and (2) *α*, a factor multiplied to ∑_1_ to increase multivariate variability and assessed the power as a function of these two parameters. We set the sample size of the SCZ group equals to that of the CTL group. We assume that the gene expression levels of the gene set obey a multivariate normal distribution (MVN). The simulations were done as follows:

Step 1: We calculated the mean vector *μ*_0_ and covariance matrix ∑_0_ from the expression values of the real gene set in CTL, and *μ*_1_ and ∑_1_ in SCZ.

Step 2: We generated the simulated expression data of CTL samples from *MVN*(*μ*_0_, ∑_0_). We generated the expression data of SCZ samples from *MVN*(*μ*_1_, (1 + *e*^*l*^)∑_1_), where *l* obeys uniform distribution (0, *α*).

Step 3: We set *α* = 1, 1.2, … , 5 and the sample size *n* = 100, 200, … , 2000. For each pair of the two parameters, we simulated expression data matrix X_1_ for SCZ. Accordingly, we simulated the expression matrix X_0_ for CTL. For samples in the data X_1_, we calculated their MD to the group centroid and obtained the vector *D*_1_ for SCZ. In the same way, we obtained the vector *D*_0_ for CTL. We used ANOVA to compare the difference between *D*_1_ and *D*_0_. For each pair of *α* and *n*, this process was repeated 100 times and the number of ANOVA *p* < 0.01 was recorded as the probability that the test correctly rejects the null hypothesis. We used the same procedure described above to perform the power analysis for the modified version of the MD-based Anderson06 test.

### Identification of SCZ-specific evQTLs

The genotype data of the SCZ and CTL samples was obtained from the CMC Knowledge Portal web site (see Data availability). For each SNP, the MAF is calculated for SCZ and CTL samples separately. The SNPs with both MAF > 0.15 were retained for the analysis. B–F tests were conducted with three genotype groups of each SNP to examine whether there is a significant difference in expression variances between any two groups. The B–F test was conducted for SCZ and CTL samples separately. The SCZ-specific evQTLs were called when the *p* < 1e−7 in SCZ samples and *p* > 0.05 in CTL samples; the CTL-specific evQTLs were called when the *p* < 1e−7 in CTL samples and *p* > 0.05 in SCZ samples. SZGR 2.0 (ref. ^99^) was used to identify antipsychotic drugs whose targets are evQTL genes.

### Reporting summary

Further information on research design is available in the Nature Research Reporting Summary linked to this article.

## Supplementary information

Supplemental information

NR reporting summary

## Data Availability

The main data set used for the analyses in this study was generated as part of the CMC supported by funding from Takeda Pharmaceuticals Company Limited, F Hoffman-La Roche and NIH Grants R01MH085542, R01MH093725, P50MH066392, P50MH080405, R01MH097276, RO1-MH-075916, P50M096891, P50MH084053S1, R37MH057881 and R37MH057881S1, HHSN271201300031C, AG02219, AG05138, and MH06692. The processed expression data matrix can be obtained from the CMC Knowledge Portal web page at https://www.synapse.org/#!Synapse:syn5609491. The file name is ‘CMC_MSSM-Penn-Pitt_DLPFC_mRNA_IlluminaHiSeq2500_gene-adjustedSVA-dataNormalization-noAncestry-adjustedLogCPM’, which is the one used for eQTL identification in the original CMC study^29^. The genotype data of SCZ and CTL samples is available at https://www.synapse.org//#!Synapse:syn3275211.

## Code availability

Source code to deterministically generate all results and plots in this paper can be found at https://github.com/cailab-tamu/scz_ge_dispersion.
