# Supplementary material for: Overdispersed gene expression in schizophrenia
Source: NPJ Schizophr. 2020 Apr 3;6:9. doi: 10.1038/s41537-020-0097-5 (PMC7125213; doi:10.1038/s41537-020-0097-5)
Supplement: Supplementary file 1 — Supplemental information [file 41537_2020_97_MOESM1_ESM.pdf]

Supplementary Information

Overdispersed gene expression in schizophrenia

Guangzao Huang[1,2,§,†], Daniel Osorio[3,§], Jinting Guan[1,2], Guoli Ji[1,2,4,*] and James J. Cai[3,5,6,*]

[1] Department of Automation, Xiamen University, Xiamen 361005, China

[2] National Institute for Data Science in Health and Medicine, Xiamen University, Xiamen 361005, China

[3] Department of Veterinary Integrative Biosciences, Texas A&M University, College Station, TX 77843, USA

[4] Innovation Center for Cell Signaling Network, Xiamen University, Xiamen 361005, China

[5] Department of Electrical and Computer Engineering, Texas A&M University, College Station, TX 77843, USA

[6] Interdisciplinary Program of Genetics, Texas A&M University, College Station, TX 77843, USA

[§] These authors contributed equally to this paper

[†] The current address of this author: College of Electrical and Electronic Engineering, Wenzhou University, Wenzhou 325035, China

[*] Corresponding authors:

Guoli Ji - Department of Automation, Xiamen University, Xiamen 361005, China. Email: glji@xmu.edu.cn

James J. Cai - Department of Veterinary Integrative Biosciences, Texas A&M University, College Station, TX 77843, USA. Email: jcai@tamu.edu

Table of Contents

Supplementary Figures. 3

Supplementary Figure 1. 3

Supplementary Figure 2. 5

Supplementary Figure 3. 6

Supplementary Figure 4. 7

Supplementary Figure 5. 11

Supplementary Figure 6. 12

Supplementary Tables. 14

Supplementary Table 1. 14

Supplementary Table 2. 16

Supplementary Table 3. 18

Supplementary Table 4. 19

Supplementary Table 5. 20

Supplementary Figures.

Supplementary Figure 1. Expression profiles for selected genes with expression variance in SCZ are significantly different from those in CTL (FDR<0.05).

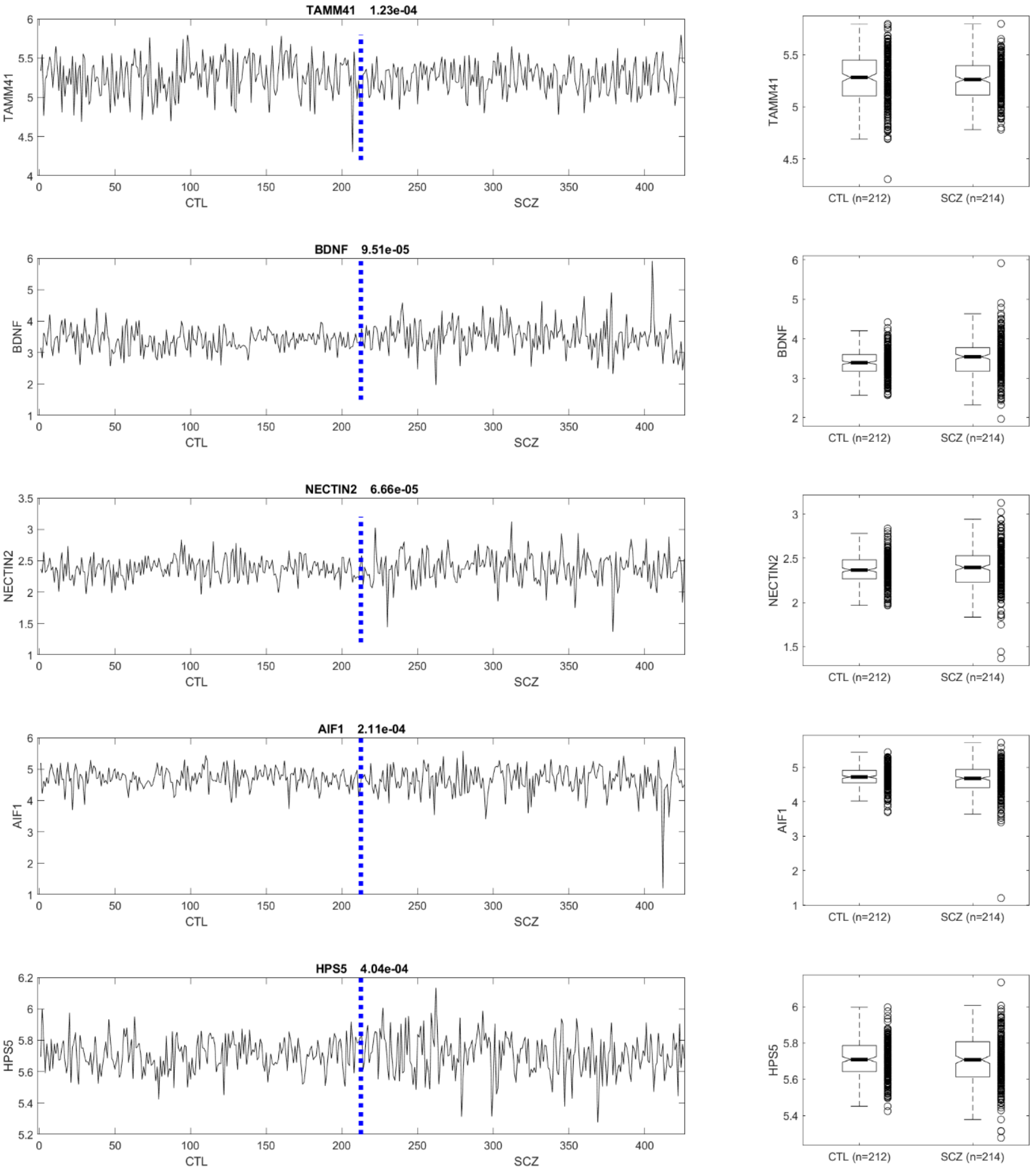

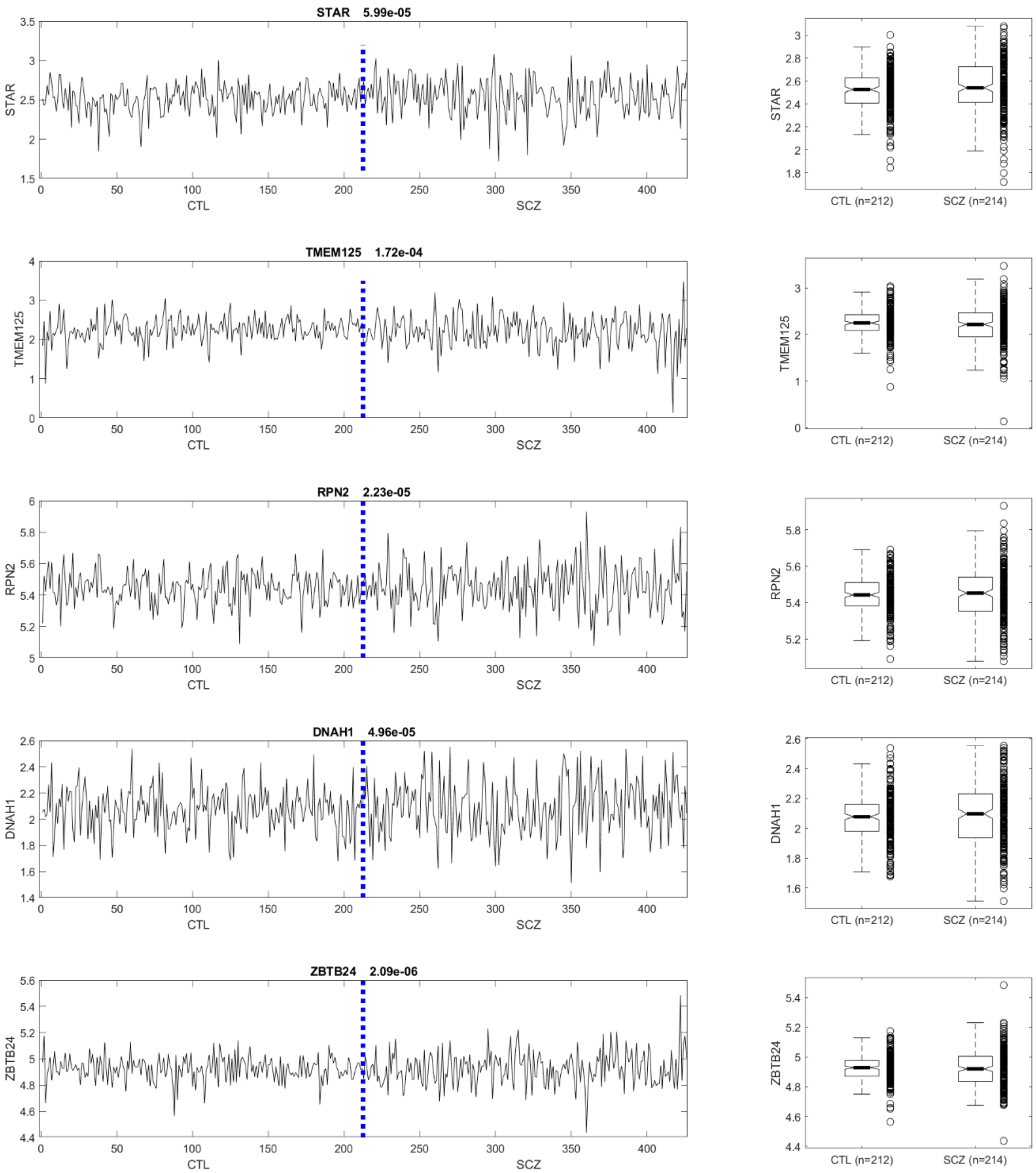
STAR 5.99e-05
TMEM125 1.72e-04
RPN2 2.23e-05
DNAH1 4.96e-05
ZBTB24 2.09e-06
STAR
TMEM125
RPN2
DNAH1
ZBTB24
CTL
SCZ
CTL (n=212)
SCZ (n=214)

Supplementary Figure 2. Removing 'outlier' samples in the SCZ group does not cause a substantial change in the significance level of the B–F test. (**a**) Red arrows indicate the two outliers in the original SCZ group that are removed in the secondary analysis. (**b**). The result of secondary analysis showing the significance remains after the two outliers are removed. The overall variance in the SCZ group is significantly greater than that in the CTL group.

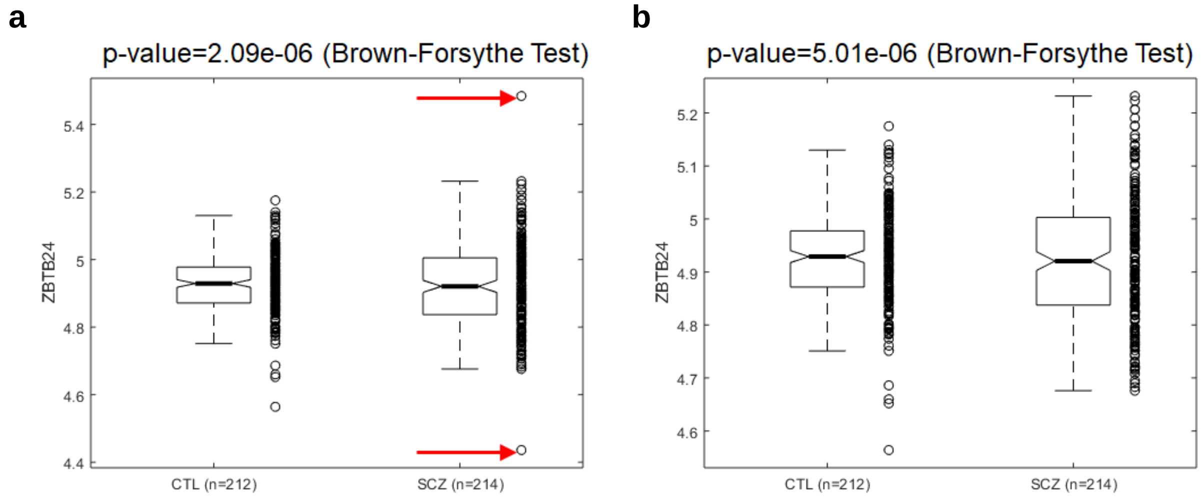

Supplementary Figure 3. Tests for equal variances in gene expression between SCZ subjects with and without antipsychotics or antidepressants treatment. (**a**) QQ plot of -log(P_1) from real data against -log(P_0) from randomly shuffled data. P_1 and P_0 are obtained using the B–F test. For each gene, the B-F test is performed to compare expression variances between SCZ subjects with and without antipsychotics treatment. To build the background distribution of B–F test *p*-values, original data (the labels of with and without antipsychotics treatment) is shuffled randomly, following the B–F test for each of genes. (**b**) Same as (**A**) but for antidepressants. (**c**) and (**d**) are the same as (**a**) and (**b**), respectively, but the test used in (**c**) and (**d**) is Kolmogorov–Smirnov (K–S) test to examine whether gene expression levels in treated and untreated SCZ patients follow the same distribution. All QQ plots show the top 750 genes whose expression variance in SCZ subjects is significantly higher than that in CTL subjects.

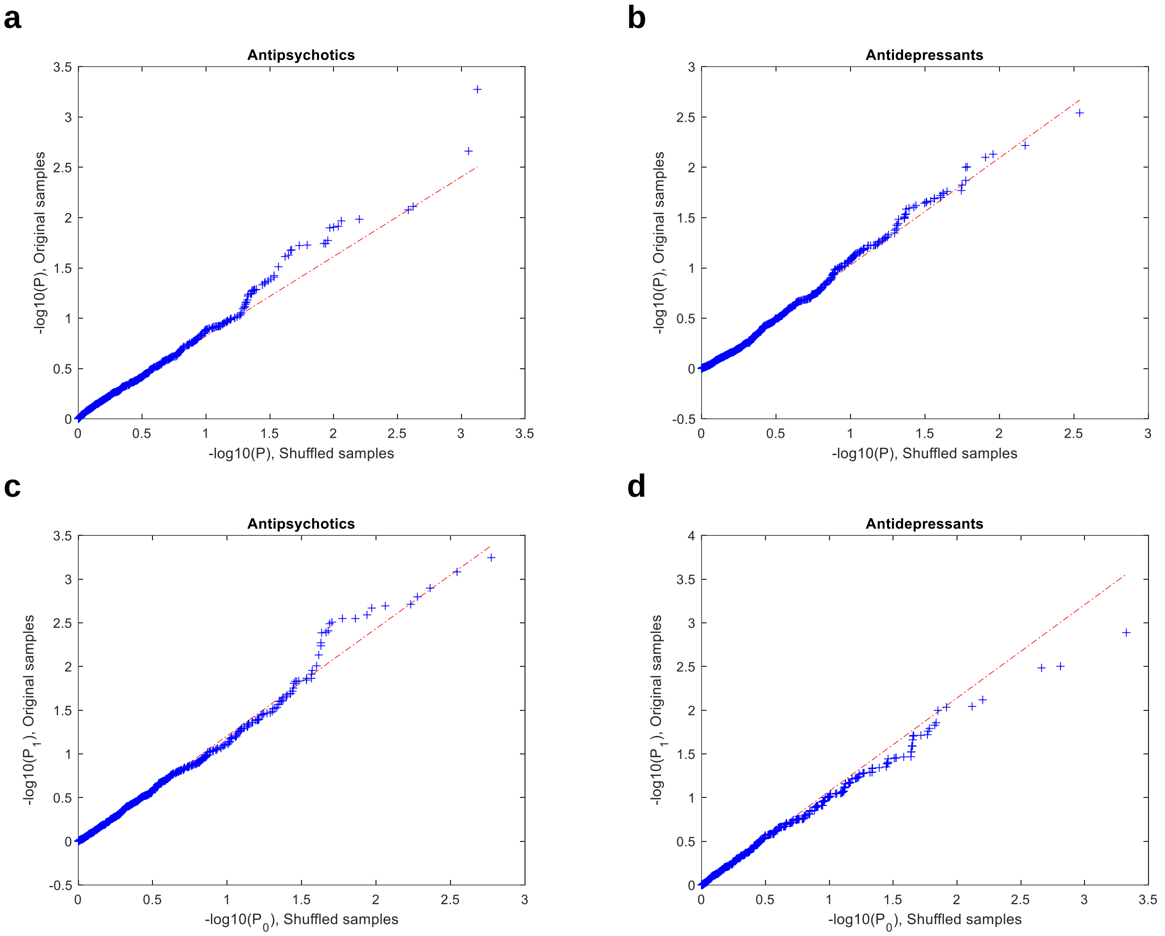

Supplementary Figure 4. Effects of the use of antipsychotics and antidepressants on expression variability of *VEGFA* and other selected genes in patients affected with schizophrenia (SCZ). The expression data is obtained from the BrainSeq project, which contains information for 320 control subjects and 175 SCZ patients.

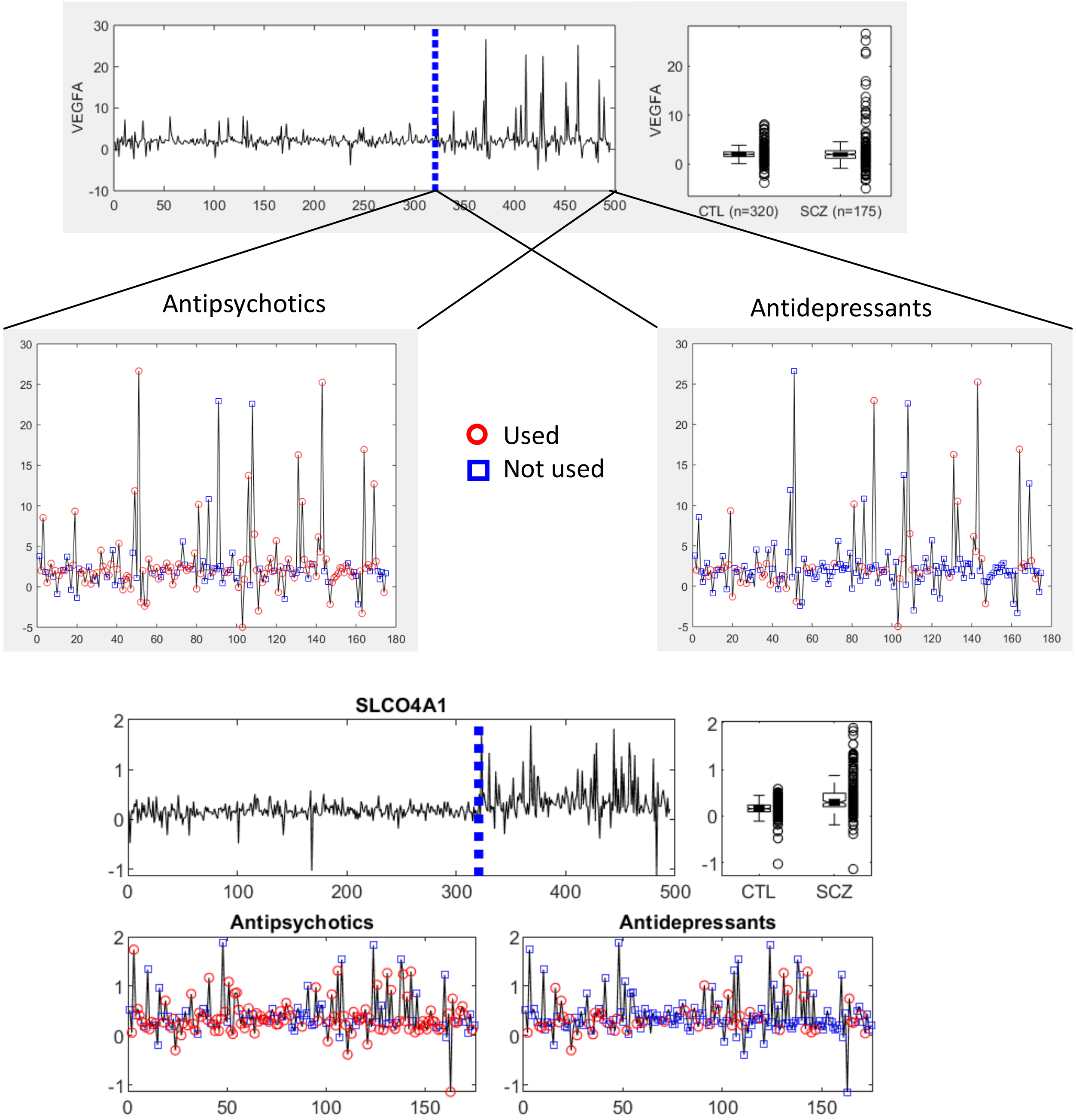

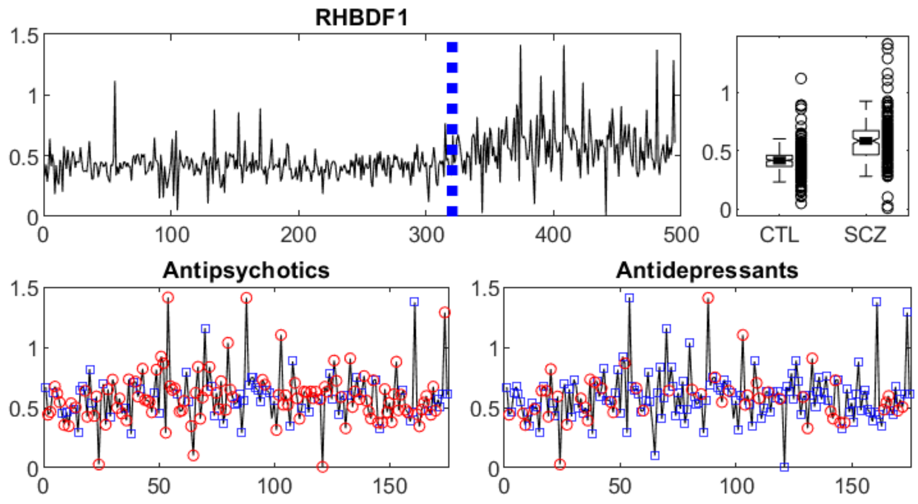
RHBDF1
CTL
SCZ
Antipsychotics
Antidepressants

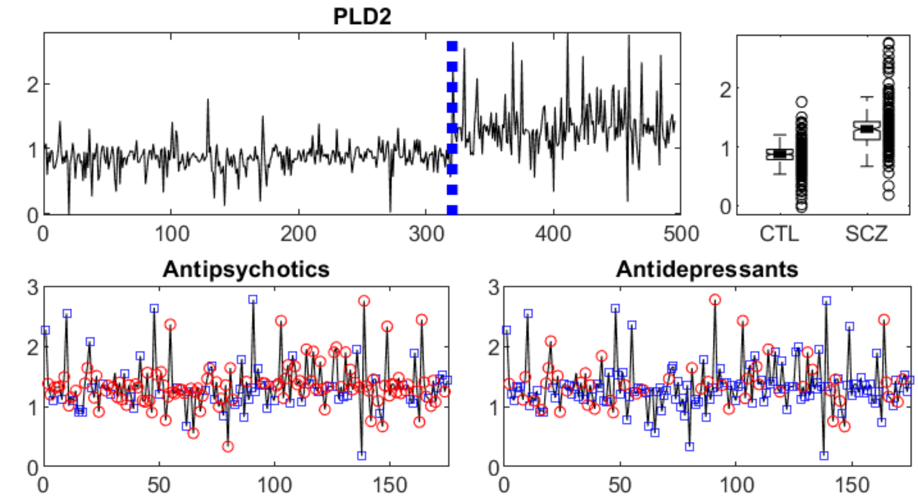
PLD2
CTL
SCZ
Antipsychotics
Antidepressants

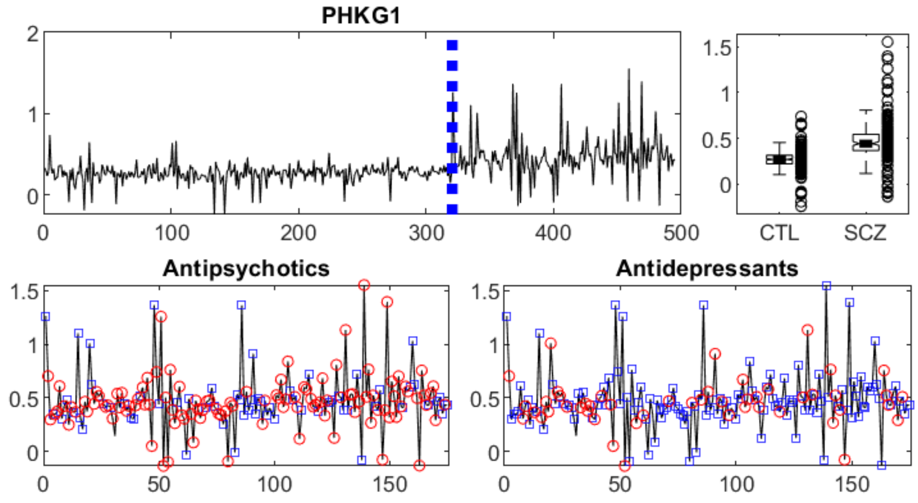
PHKG1
CTL
SCZ
Antipsychotics
Antidepressants

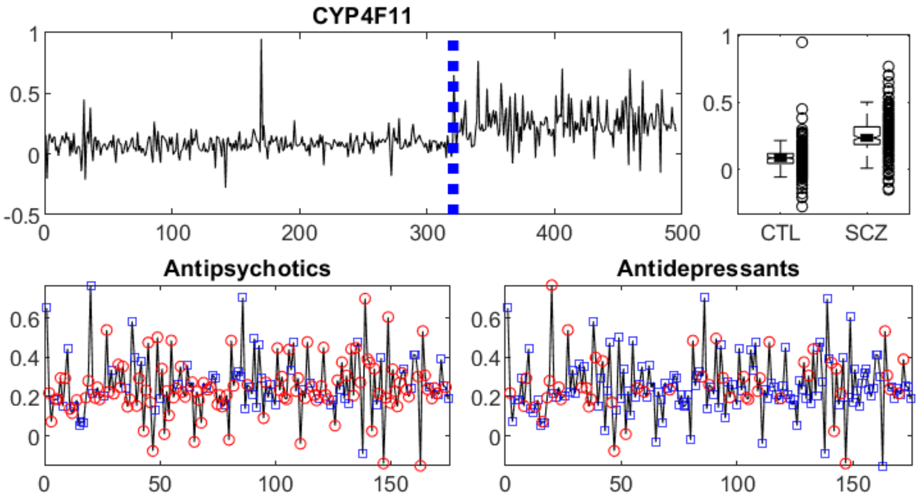
CYP4F11
CTL
SCZ
Antipsychotics
Antidepressants

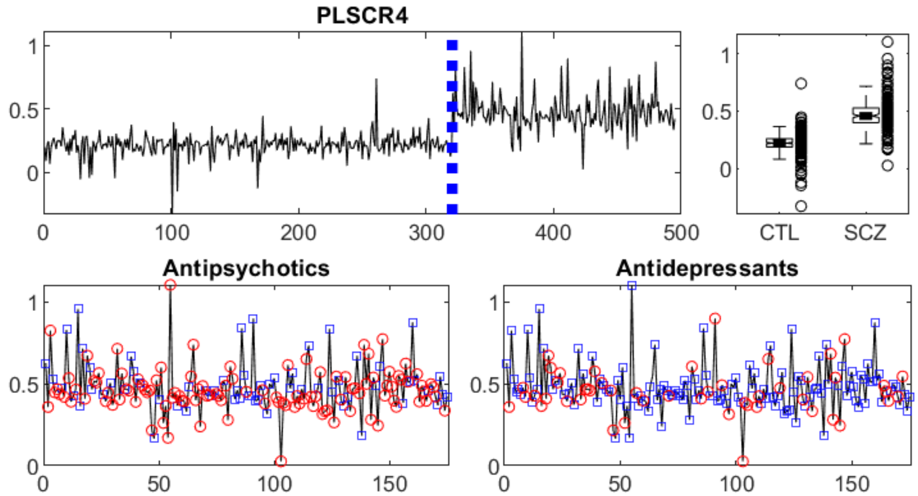
PLSCR4
CTL
SCZ
Antipsychotics
Antidepressants

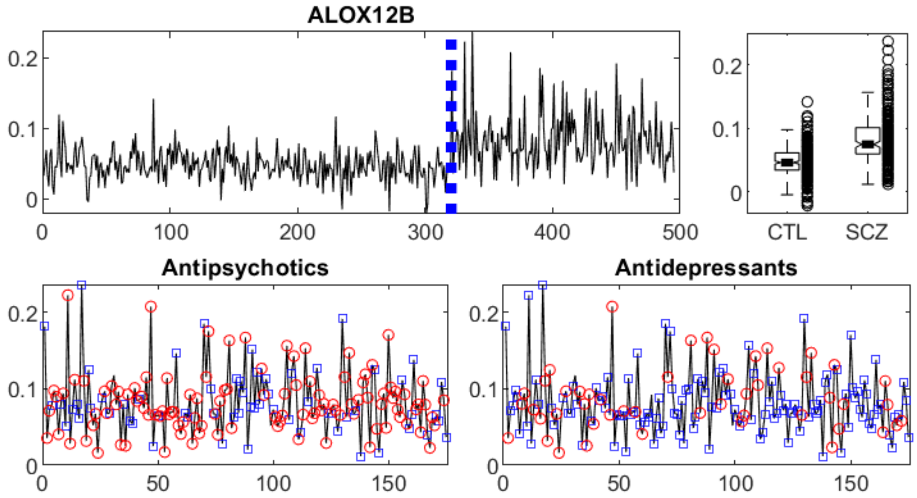
ALOX12B
CTL
SCZ
Antipsychotics
Antidepressants

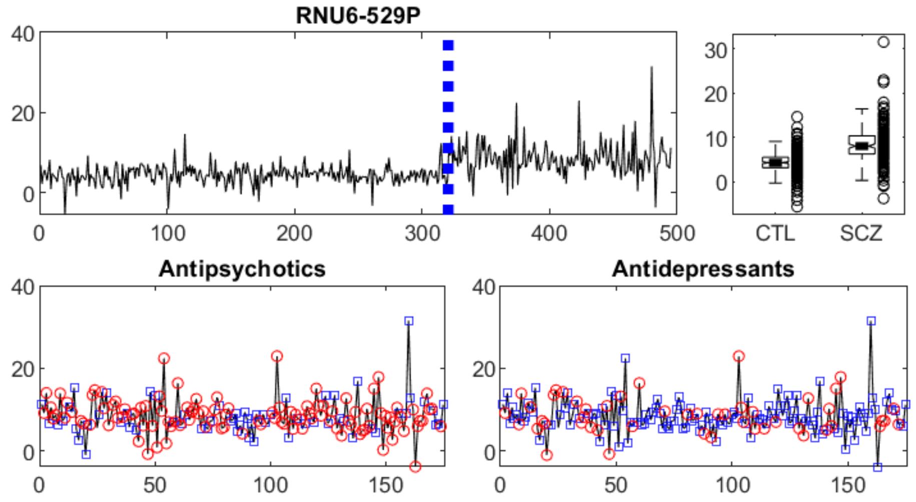
RNU6-529P
CTL
SCZ
Antipsychotics
Antidepressants

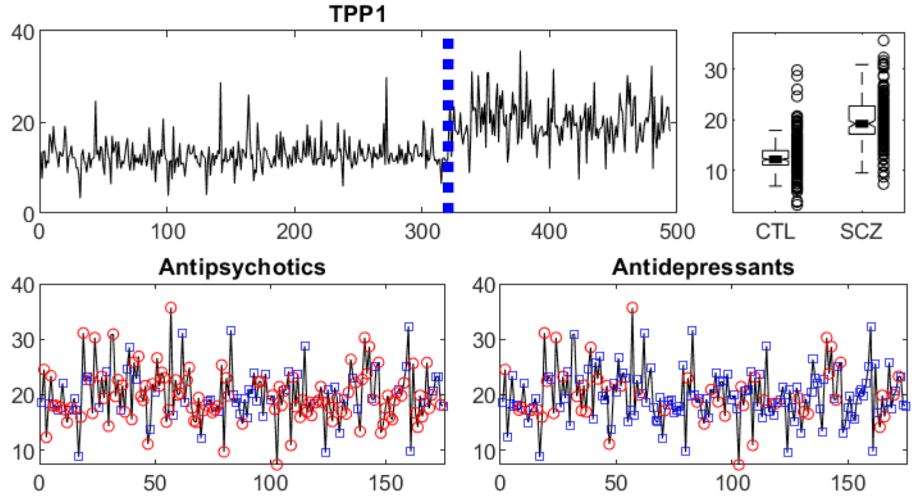
TPP1
CTL
SCZ
Antipsychotics
Antidepressants

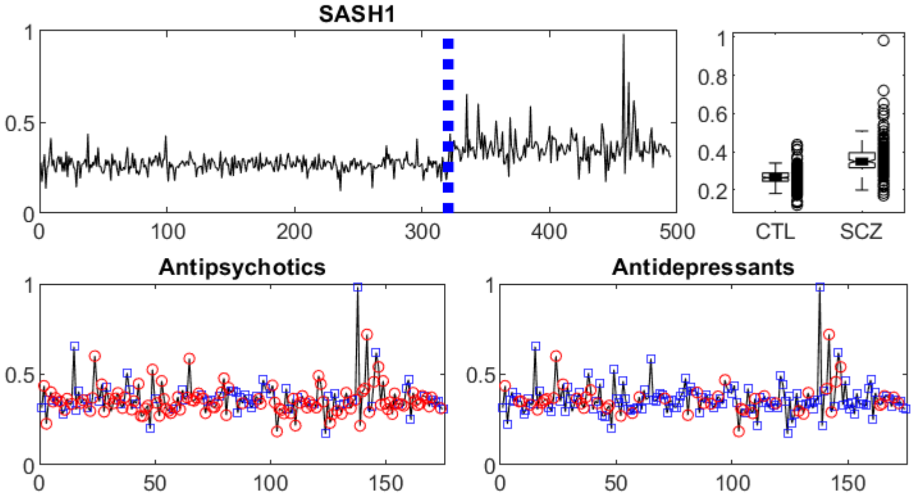
SASH1
CTL
SCZ
Antipsychotics
Antidepressants

Supplementary Figure 5. Statistical power of the MD-based Anderson (2006) test. (**a**) and our modified version of test (**b**). The power is estimated using simulated data in the context of detecting significant heterogeneity in multivariate gene expression between two groups. Each contour line depicts the power, i.e., the likelihood level (%) that a random data set would yield a chance finding, as a function of the sample size per group, and alpha—a factor for introducing variance heterogeneity to one of the groups.

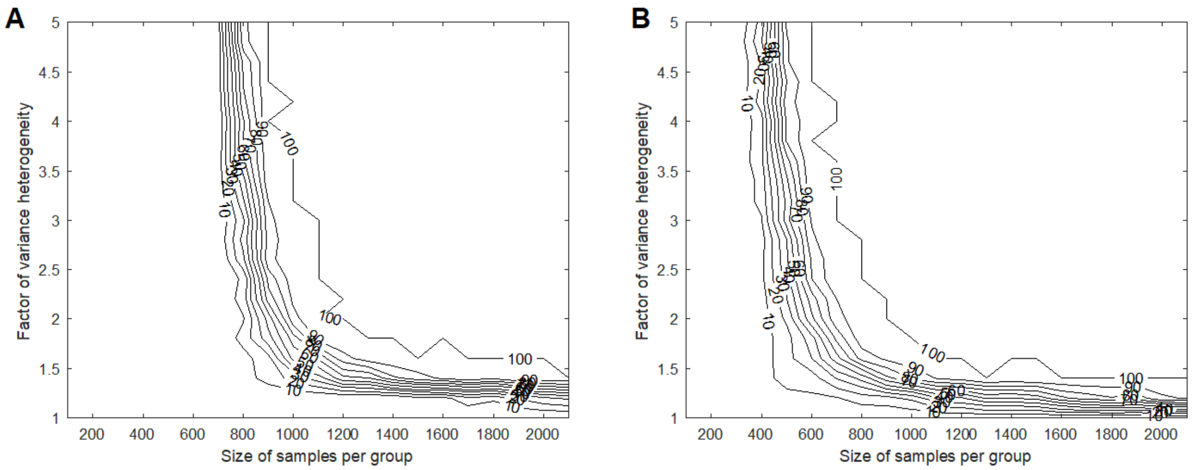

Supplementary Figure 6. Additional SCZ-specific evQTL examples.

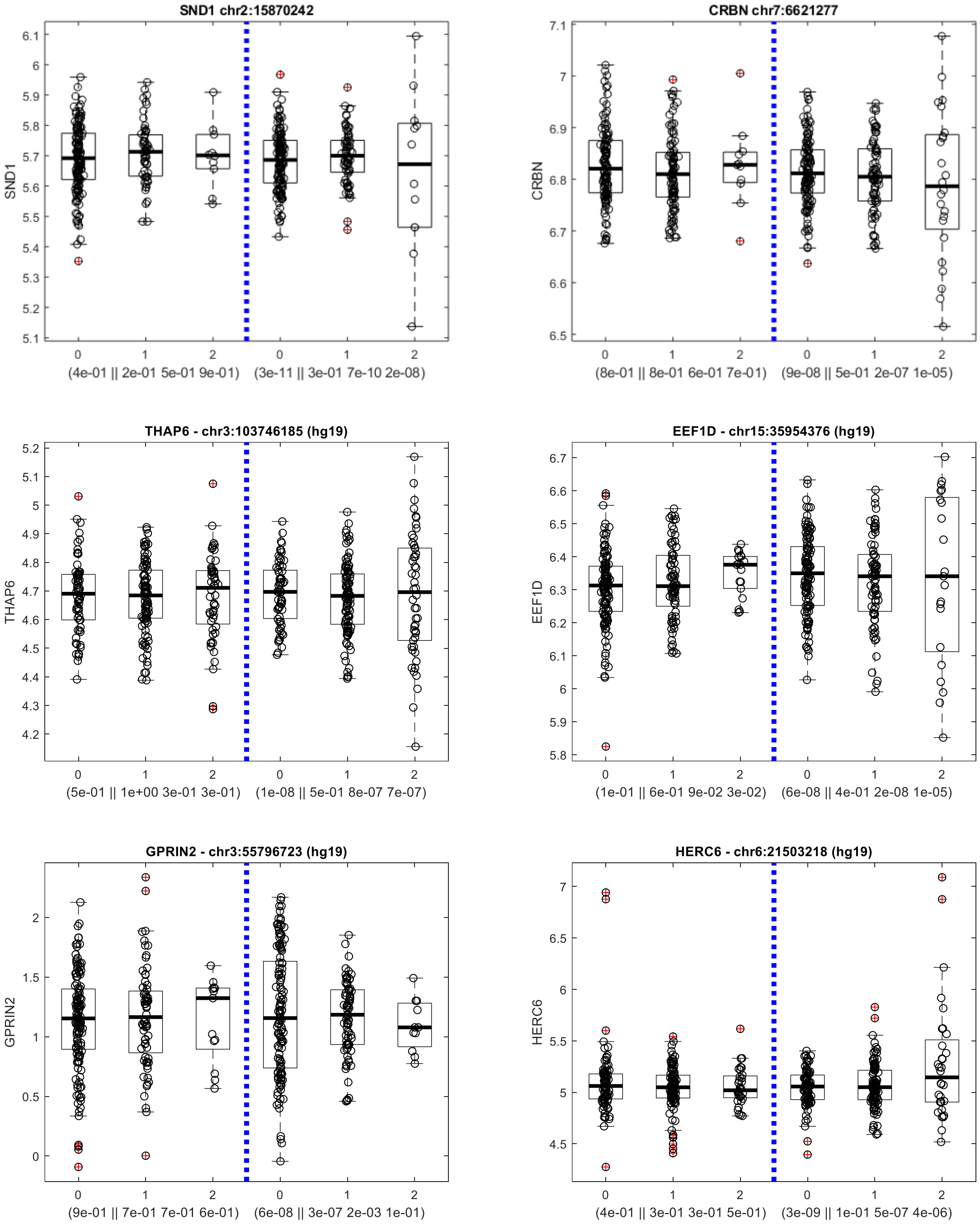

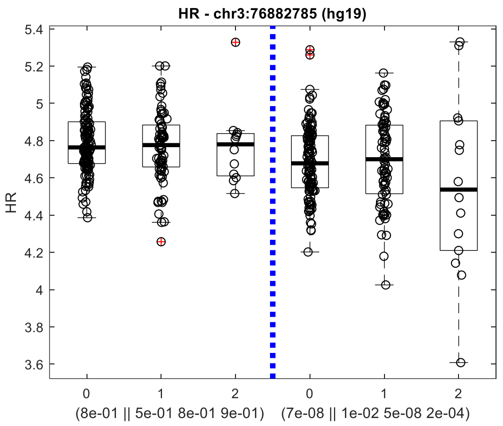
HR - chr3:76882785 (hg19)
HR
0 1 2 0 1 2
(8e-01 || 5e-01 8e-01 9e-01) (7e-08 || 1e-02 5e-08 2e-04)

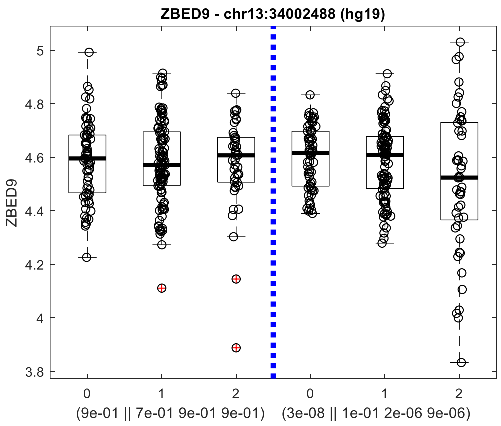
ZBED9 - chr13:34002488 (hg19)
ZBED9
0 1 2 0 1 2
(9e-01 || 7e-01 9e-01 9e-01) (3e-08 || 1e-01 2e-06 9e-06)

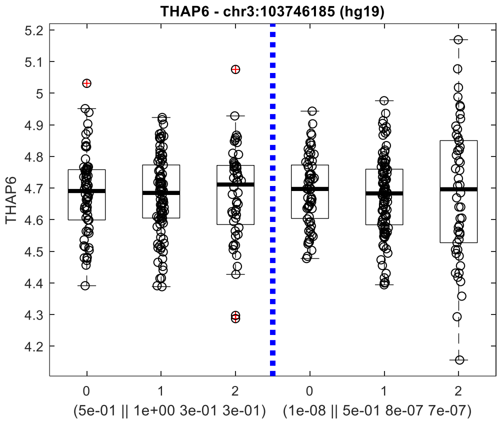
THAP6 - chr3:103746185 (hg19)
THAP6
0 1 2 0 1 2
(5e-01 || 1e+00 3e-01 3e-01) (1e-08 || 5e-01 8e-07 7e-07)

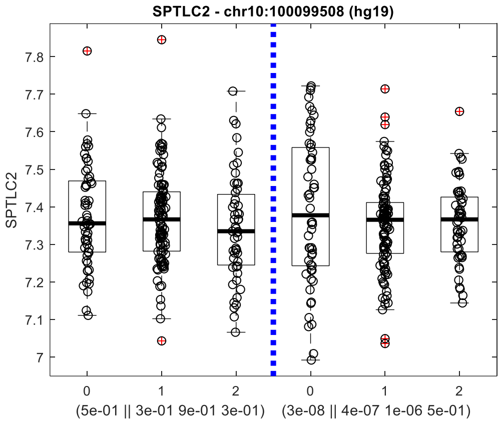
SPTLC2 - chr10:100099508 (hg19)
SPTLC2
0 1 2 0 1 2
(5e-01 || 3e-01 9e-01 3e-01) (3e-08 || 4e-07 1e-06 5e-01)

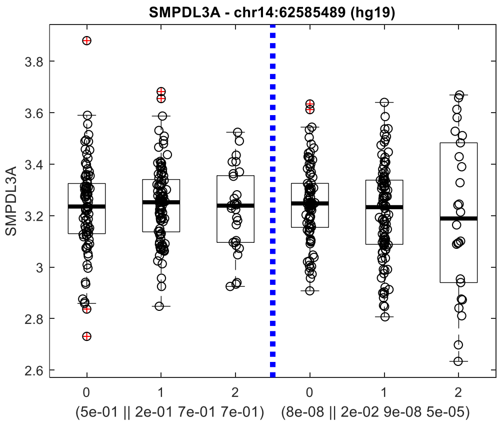
SMPDL3A - chr14:62585489 (hg19)
SMPDL3A
0 1 2 0 1 2
(5e-01 || 2e-01 7e-01 7e-01) (8e-08 || 2e-02 9e-08 5e-05)

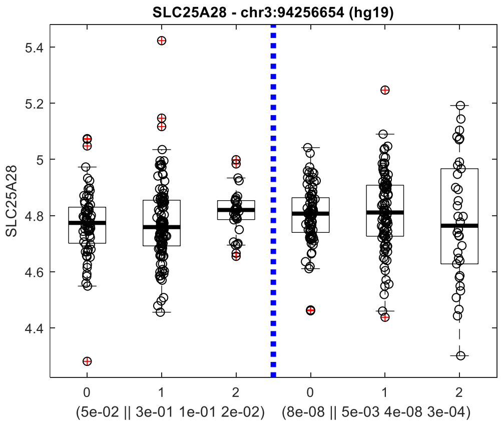
SLC25A28 - chr3:94256654 (hg19)
SLC25A28
0 1 2 0 1 2
(5e-02 || 3e-01 1e-01 2e-02) (8e-08 || 5e-03 4e-08 3e-04)

Supplementary Tables.

Supplementary Table 1. List of 88 differentially variable (DV) genes whose expression variance in SCZ is significantly different from that in CTL (FDR<0.05). For each gene, *p*-value of Brown–Forsythe (B-F) test, comparison between expression standard deviation (Std) in SCZ and CTL, and gene name, are given.

**Gene Symbol**	**P-value**	**Std(SCZ)> Std(CTL)?**	**HGNC Gene Name**
ZBTB24	2.09E-06	Yes	zinc finger and BTB domain containing 24
RALY	5.07E-06	Yes	RALY heterogeneous nuclear ribonucleoprotein
BICDL1	8.52E-06	Yes	BICD family like cargo adaptor 1
AARS	1.54E-05	Yes	alanyl-tRNA synthetase
RPN2	2.23E-05	Yes	ribophorin II
LCORL	2.30E-05	Yes	ligand dependent nuclear receptor corepressor like
NPEPL1	2.36E-05	Yes	aminopeptidase like 1
CNP	3.33E-05	Yes	2,3-cyclic nucleotide 3 phosphodiesterase
MAGEF1	4.31E-05	Yes	MAGE family member F1
TOM1	4.58E-05	Yes	target of myb1 membrane trafficking protein
LUC7L	4.74E-05	Yes	LUC7 like
DNAH1	4.96E-05	Yes	dynein axonemal heavy chain 1
RNF125	5.38E-05	Yes	ring finger protein 125
STAR	5.99E-05	Yes	steroidogenic acute regulatory protein
EXOC1	6.03E-05	Yes	exocyst complex component 1
NECTIN2	6.66E-05	Yes	nectin cell adhesion molecule 2
GALT	6.84E-05	Yes	galactose-1-phosphate uridylyltransferase
CCR10	7.39E-05	Yes	C-C motif chemokine receptor 10
BDNF	9.51E-05	Yes	brain derived neurotrophic factor
SIRT7	9.72E-05	Yes	sirtuin 7
PLPP2	1.04E-04	Yes	phospholipid phosphatase 2
NUDT14	1.09E-04	Yes	nudix hydrolase 14
TTC37	1.19E-04	Yes	tetratricopeptide repeat domain 37
ZNF33B	1.28E-04	Yes	zinc finger protein 33B
INTS11	1.29E-04	Yes	integrator complex subunit 11
B2M	1.43E-04	Yes	beta-2-microglobulin
HR	1.45E-04	Yes	HR, lysine demethylase and nuclear receptor corepressor
MARCH9	1.46E-04	Yes	membrane associated ring-CH-type finger 9
NQO1	1.47E-04	Yes	NAD(P)H quinone dehydrogenase 1
OPN3	1.49E-04	Yes	opsin 3
CARS2	1.57E-04	Yes	cysteinyl-tRNA synthetase 2, mitochondrial
TMEM125	1.72E-04	Yes	transmembrane protein 125
PNPLA8	1.84E-04	Yes	patatin like phospholipase domain containing 8
CX3CR1	1.96E-04	Yes	C-X3-C motif chemokine receptor 1
ATP5F1C	2.04E-04	Yes	ATP synthase F1 subunit gamma
STOML1	2.08E-04	Yes	stomatin like 1
EIF2D	2.09E-04	Yes	eukaryotic translation initiation factor 2D
TDG	2.10E-04	Yes	thymine DNA glycosylase
AIF1	2.11E-04	Yes	allograft inflammatory factor 1
BORCS6	2.16E-04	Yes	BLOC-1 related complex subunit 6
ADAMTS9	2.20E-04	Yes	ADAM metallopeptidase with thrombospondin type 1 motif 9
RTN4	2.27E-04	Yes	reticulon 4
HERPUD2	2.40E-04	Yes	HERPUD family member 2
DHPS	2.43E-04	Yes	deoxyhypusine synthase
RPS14	2.44E-04	Yes	ribosomal protein S14
CCNL1	2.48E-04	Yes	cyclin L1
LAP3	2.51E-04	Yes	leucine aminopeptidase 3
AGPAT2	2.57E-04	Yes	1-acylglycerol-3-phosphate O-acyltransferase 2
RBM11	2.59E-04	Yes	RNA binding motif protein 11
SERP2	2.62E-04	Yes	stress associated endoplasmic reticulum protein family member 2
CYSLTR2	2.79E-04	Yes	cysteinyl leukotriene receptor 2
GPRASP2	2.79E-04	Yes	G protein-coupled receptor associated sorting protein 2

NLE1	2.81E-04	Yes	notchless homolog 1
REXO2	2.90E-04	Yes	RNA exonuclease 2
GLTP	3.15E-04	Yes	glycolipid transfer protein
VEGFA	3.33E-04	Yes	vascular endothelial growth factor A
MAP2K4	3.39E-04	Yes	mitogen-activated protein kinase kinase 4
PLP1	3.50E-04	Yes	proteolipid protein 1
TMEM235	3.55E-04	Yes	transmembrane protein 235
KCNJ16	3.59E-04	Yes	potassium voltage-gated channel subfamily J member 16
NEK10	3.74E-04	Yes	NIMA related kinase 10
FAM84B	3.75E-04	Yes	family with sequence similarity 84 member B
C1S	3.78E-04	Yes	complement C1s
JAM2	3.90E-04	Yes	junctional adhesion molecule 2
CHGA	3.94E-04	Yes	chromogranin A
CAB39L	3.97E-04	Yes	calcium binding protein 39 like
HPS5	4.04E-04	Yes	HPS5, biogenesis of lysosomal organelles complex 2 subunit 2
NCKAP1L	4.09E-04	Yes	NCK associated protein 1 like
NQO2	4.11E-04	Yes	N-ribosyldihydronicotinamide:quinone reductase 2
MRPS17	4.19E-04	Yes	mitochondrial ribosomal protein S17
C14orf80	4.23E-04	Yes	C14orf80
STRN	4.35E-04	Yes	striatin
VWA2	4.61E-04	Yes	von Willebrand factor A domain containing 2
OLFML3	4.65E-04	Yes	olfactomedin like 3
RASGRP3	4.86E-04	Yes	RAS guanyl releasing protein 3
TUBGCP6	5.08E-04	Yes	tubulin gamma complex associated protein 6
CDC27	5.35E-04	Yes	cell division cycle 27
CRCP	5.42E-04	Yes	CGRP receptor component
CHTF18	5.45E-04	Yes	chromosome transmission fidelity factor 18
UNC93B1	5.54E-04	Yes	unc-93 homolog B1, TLR signaling regulator
DIS3	5.55E-04	Yes	DIS3 homolog, exosome endoribonuclease and 3-5 exoribonuclease
GDF7	5.57E-04	Yes	growth differentiation factor 7
NDC1	5.63E-04	Yes	NDC1 transmembrane nucleoporin
ZFP36L2	5.82E-04	Yes	ZFP36 ring finger protein like 2
GNPDA1	5.84E-04	Yes	glucosamine-6-phosphate deaminase 1
LURAP1	5.87E-04	Yes	leucine rich adaptor protein 1
MRPS5	6.06E-04	Yes	mitochondrial ribosomal protein S5
TAMM41	1.23E-04	No	TAM41 mitochondrial translocator assembly and maintenance homolog

Supplementary Table 2. List of 110 differentially variable (DV) gene sets whose expression variance in SCZ is higher than that in CTL (P-value < 0.05). For each gene set, the number of genes in the gene set and the number of genes used in the test are given.

Gene Set Name	Genes used	Set Size	p-value
GO_PHOSPHATIDYLINOSITOL_3_KINASE_COMPLEX	15	20	7.47E-05
GO_REGULATION_OF_B_CELL_PROLIFERATION	28	55	0.000128
GO_CEREBELLAR_CORTEX_MORPHOGENESIS	23	30	0.000185
GO_REGULATION_OF_ENDOPLASMIC_RETICULUM_UNFOLDED_PROTEIN_RESPONSE	22	28	0.000248
GO_GENETIC_IMPRINTING	15	20	0.000442
GO_POSITIVE_REGULATION_OF_STRIATED_MUSCLE_CELL_DIFFERENTIATION	24	52	0.000544
GO_ENDORIBONUCLEASE_ACTIVITY	33	54	0.000557
GO_INSULIN_LIKE_GROWTH_FACTOR_RECEPTOR_SIGNALING_PATHWAY	14	14	0.000787
GO_LIPOPROTEIN_PARTICLE_RECEPTOR_ACTIVITY	12	16	0.000976
GO_RNA_POLYMERASE_II_ACTIVATING_TRANSCRIPTION_FACTOR_BINDING	24	36	0.000983
GO_FOLIC_ACID_CONTAINING_COMPOUND_METABOLIC_PROCESS	22	29	0.001096
GO_CEREBELLAR_CORTEX_FORMATION	19	22	0.00174
GO_NEGATIVE_REGULATION_OF_VASCULATURE_DEVELOPMENT	40	80	0.001791
GO_RETROGRADE_TRANSPORT_VESICLE_RECYCLING_WITHIN_GOLGI	19	24	0.001965
GO_NEUROMUSCULAR_JUNCTION_DEVELOPMENT	28	36	0.002129
GO_CEREBELLAR_PURKINJE_CELL_LAYER_DEVELOPMENT	20	24	0.002393
GO_NEGATIVE_REGULATION_OF_EPITHELIAL_CELL_APOPTOTIC_PROCESS	18	35	0.003546
GO_PYRIMIDINE_DEOXYRIBONUCLEOTIDE_METABOLIC_PROCESS	14	18	0.004017
GO_NEGATIVE_REGULATION_OF_CIRCADIAN_RHYTHM	11	17	0.004143
GO_NEGATIVE_REGULATION_OF_OXIDOREDUCTASE_ACTIVITY	13	26	0.004287
GO_ACTIVATING_TRANSCRIPTION_FACTOR_BINDING	40	57	0.004511
GO_FOUR_WAY_JUNCTION_DNA_BINDING	13	15	0.004971
GO_CELL_DIFFERENTIATION_IN_HINDBRAIN	15	21	0.005239
GO_CEREBELLAR_PURKINJE_CELL_LAYER_MORPHOGENESIS	11	14	0.005589
GO_ESTABLISHMENT_OF_MITOTIC_SPINDLE_ORIENTATION	16	20	0.006199
GO_PTERIDINE_CONTAINING_COMPOUND_METABOLIC_PROCESS	28	36	0.006637
GO_HINDBRAIN_MORPHOGENESIS	29	40	0.007094
GO_REGULATION_OF_ASTROCYTE_DIFFERENTIATION	19	27	0.007418
GO_PHOSPHATIDYLCHOLINE_BIOSYNTHETIC_PROCESS	18	27	0.008391
GO_REGULATION_OF_GENE_EXPRESSION_BY_GENETIC_IMPRINTING	12	16	0.00853
GO_METALLOEXOPEPTIDASE_ACTIVITY	26	53	0.008896
GO_MAMMARY_GLAND_EPITHELIUM_DEVELOPMENT	27	53	0.009186
GO_LOW_DENSITY_LIPOPROTEIN_RECEPTOR_ACTIVITY	11	13	0.009581
GO_REGULATION_OF_IRE1_MEDIATED_UNFOLDED_PROTEIN_RESPONSE	11	13	0.009716
GO_AORTA_MORPHOGENESIS	13	22	0.010558
GO_PONS_DEVELOPMENT	9	11	0.010597
GO_PHOSPHATIDYLSERINE_ACYL_CHAIN_REMODELING	5	17	0.011621
GO_CYCLIC_NUCLEOTIDE_METABOLIC_PROCESS	36	57	0.011906
GO_NEGATIVE_REGULATION_OF_NECROTIC_CELL_DEATH	8	11	0.012233
GO_ALCOHOL_TRANSMEMBRANE_TRANSPORTER_ACTIVITY	11	24	0.012605
GO_BETA_TUBULIN_BINDING	34	36	0.013027
GO_ASYMMETRIC_PROTEIN_LOCALIZATION	16	19	0.014989
GO_REGULATION_OF_NECROPTOTIC_PROCESS	8	11	0.015032
GO_ENTRAINMENT_OF_CIRCADIAN_CLOCK	20	26	0.015481
GO_LUNG_ALVEOLUS_DEVELOPMENT	26	41	0.015732
GO_REGULATION_OF_MYOBLAST_DIFFERENTIATION	23	48	0.016142
GO_RESPONSE_TO_MONOAMINE	30	35	0.016686
GO_NEURON_PROJECTION_EXTENSION	42	53	0.017939
GO_REGULATION_OF_PROTEIN_GLYCOSYLATION	10	14	0.018605
GO_MISFOLDED_OR_INCOMPLETELY_SYNTHESIZED_PROTEIN_CATABOLIC_PROCESS	15	16	0.019102
GO_LIGAND_DEPENDENT_NUCLEAR_RECEPTOR_BINDING	21	23	0.019312
GO_REGULATION_OF_CELLULAR_SENESCENCE	19	26	0.019314
GO_RESPONSE_TO_INTERFERON_GAMMA	67	144	0.019637
GO_ADENOSINE_DEAMINASE_ACTIVITY	6	11	0.01969
GO_RESPONSE_TO_COBALT_ION	11	13	0.019726
GO_ANATOMICAL_STRUCTURE_MATURATION	28	39	0.020119

GO_CYSTEINE_TYPE_ENDOPEPTIDASE_INHIBITOR_ACTIVITY_INVOLVED_IN_APOPTOTIC_PROCESS	13	23	0.020416
GO_SPERM_FLAGELLUM	31	57	0.020598
GO_ICOSANOID_BIOSYNTHETIC_PROCESS	20	46	0.020651
GO_GAMMA_TUBULIN_BINDING	20	24	0.020713
GO_NEURON_PROJECTION_REGENERATION	23	33	0.020964
GO_REGULATION_OF_HISTONE_H3_K9_METHYLATION	11	17	0.021353
GO_ENDODEOXYRIBONUCLEASE_ACTIVITY_PRODUCING_5_PHOSPHOMONOESTERS	9	12	0.021898
GO_POSITIVE_REGULATION_OF_INSULIN_SECRETION_INVOLVED_IN_CELLULAR_RESPONSE_TO_GLUCOSE_STIMULUS	16	29	0.021975
GO_HOPS_COMPLEX	13	14	0.022096
GO_RESPONSE_TO_ACIDIC_PH	9	21	0.022763
GO_CEREBRAL_CORTEX_RADIALLY_ORIENTED_CELL_MIGRATION	24	29	0.023179
GO_REGULATION_OF_RESPIRATORY_SYSTEM_PROCESS	10	16	0.023361
GO_REGULATION_OF_CARDIAC_MUSCLE_TISSUE_DEVELOPMENT	27	48	0.023983
GO_BAF_TYPE_COMPLEX	22	23	0.024045
GO_NEGATIVE_REGULATION_OF_PHOSPHOPROTEIN_PHOSPHATASE_ACTIVITY	13	15	0.025243
GO_NEGATIVE_REGULATION_OF_VIRAL_RELEASE_FROM_HOST_CELL	12	16	0.025808
GO_THYROID_HORMONE_RECEPTOR_BINDING	25	30	0.026377
GO_REGULATION_OF_STEM_CELL_POPULATION_MAINTENANCE	10	17	0.027781
GO_PEPTIDYL_CYSTEINE_MODIFICATION	17	20	0.027821
GO_DEATH_RECEPTOR_ACTIVITY	10	24	0.028411
GO_REGULATION_OF_ORGANIC_ACID_TRANSPORT	32	50	0.028846
GO_NEGATIVE_REGULATION_OF_ERAD_PATHWAY	11	12	0.029155
GO_INTRONIC_TRANSCRIPTION_REGULATORY_REGION_DNA_BINDING	8	12	0.029386
GO_REGULATION_OF_INSULIN_RECEPTOR_SIGNALING_PATHWAY	30	43	0.030568
GO_NEGATIVE_REGULATION_OF_GLIAL_CELL_DIFFERENTIATION	16	26	0.030843
GO_TRANSFERASE_ACTIVITY_TRANSFERRING_NITROGENOUS_GROUPS	14	23	0.030988
GO_ARTERY_MORPHOGENESIS	26	51	0.031398
GO_ENTEROENDOCRINE_CELL_DIFFERENTIATION	9	19	0.03298
GO_ESTABLISHMENT_OF_MITOTIC_SPINDLE_LOCALIZATION	19	24	0.034051
GO_AP_TYPE_MEMBRANE_COAT_ADAPTOR_COMPLEX	34	40	0.03454
GO_ACTIVATION_OF_JUN_KINASE_ACTIVITY	23	35	0.034652
GO_ACYL_COA_BIOSYNTHETIC_PROCESS	41	54	0.034656
GO_REGULATION_OF_ESTABLISHMENT_OF_ENDOTHELIAL_BARRIER	5	12	0.035186
GO_POSITIVE_REGULATION_OF_CELL_JUNCTION_ASSEMBLY	17	24	0.03531
GO_NEGATIVE_REGULATION_OF_CELLULAR_RESPONSE_TO_TRANSFORMING_GROWTH_FACTOR_BETA_STIMULUS	44	66	0.036344
GO_CARBOXYPEPTIDASE_ACTIVITY	17	41	0.036497
GO_REGULATION_OF_OSTEOBLAST_PROLIFERATION	17	23	0.037838
GO_PROLINE_RICH_REGION_BINDING	17	19	0.039542
GO_SEGMENTATION	51	89	0.040282
GO_PROTEIN_LOCALIZATION_TO_CYTOSKELETON	25	30	0.040566
GO_BRANCHING_INVOLVED_IN_MAMMARY_GLAND_DUCT_MORPHOGENESIS	10	20	0.040843
GO_REGULATION_OF_BLOOD_PRESSURE	68	169	0.040843
GO_POSITIVE_REGULATION_OF_MYOTUBE_DIFFERENTIATION	10	29	0.043661
GO_PROTEIN_DEALKYLATION	24	29	0.043856
GO_SOMITE_DEVELOPMENT	44	78	0.044243
GO_CORONARY_VASCULATURE_DEVELOPMENT	27	37	0.044453
GO_PEPTIDE_CATABOLIC_PROCESS	16	25	0.044801
GO_CELLULAR_PIGMENT_ACCUMULATION	7	12	0.045288
GO_CELLULAR_RESPONSE_TO_REACTIVE_NITROGEN_SPECIES	14	19	0.045555
GO_NEGATIVE_REGULATION_OF_STRIATED_MUSCLE_CELL_APOPTOTIC_PROCESS	11	17	0.045941
GO_NPBAF_COMPLEX	12	12	0.046628
GO_REGULATION_OF_TELOMERE_CAPPING	17	22	0.04801
GO_REGULATION_OF_CARDIAC_MUSCLE_CELL_PROLIFERATION	17	29	0.048426
GO_NEGATIVE_REGULATION_OF_CARTILAGE_DEVELOPMENT	10	26	0.04971

Supplementary Table 3. List of 9 differentially variable (DV) gene sets whose expression variance in SCZ is smaller than that in CTL (P-value < 0.05). For each gene set, the number of genes in the gene set and the number of genes used in the test are given.

Gene Set Name	Genes used	Set Size	P-value
GO_NEUREXIN_FAMILY_PROTEIN_BINDING	10	14	0.000303
GO_PROTEASOME_ACCESSORY_COMPLEX	23	24	0.003865
GO_PLATELET_DERIVED_GROWTH_FACTOR_RECEPTOR_BINDING	10	15	0.018709
GO_SMN_SM_PROTEIN_COMPLEX	13	17	0.018976
GO_RESPONSE_TO_VITAMIN_D	15	33	0.021413
GO_ENDODERMAL_CELL_FATE_COMMITMENT	7	13	0.022717
GO_NEURON_CELL_CELL_ADHESION	15	16	0.023955
GO_CELLULAR_RESPONSE_TO_VITAMIN	12	26	0.038477
GO_PHOSPHATE_ION_BINDING	5	11	0.046763

Supplementary Table 4. List of evQTLs.

The table is available for downloading at: https://github.com/cailab-tamu/SCZ_GE_Dispersion/blob/master/s4_scz_vqtl_detection/Suppl_table_S4.xlsx

Supplementary Table 5. Information of donors of the CommonMind project.

Individual ID	Institution	CTL or SCZ	Gender	Ethnicity	Brain Weight (g)	PMI (hrs)	pH	Year of Autops (y)	Age of Death	Height (in)	Weight (lbs)
CMC_MSSM_029	MSSM	Control	Female	Caucasian	1005	5.7	6.1	1995	82	67.00003618	180
CMC_MSSM_035	MSSM	Control	Male	Caucasian	1102	2	6.6	1994	75	NA	118
CMC_MSSM_036	MSSM	Control	Female	Caucasian	1010	3.8	6.4	1995	82	NA	121
CMC_MSSM_039	MSSM	Control	Female	Caucasian	1174	24	6.3	2004	68	69.00003726	275
CMC_MSSM_040	MSSM	Control	Male	Caucasian	1376	23.8	6.69	2005	64	NA	214
CMC_MSSM_041	MSSM	Control	Female	Caucasian	940	3.4	6.1	1986	90	NA	NA
CMC_MSSM_044	MSSM	Control	Male	Caucasian	1402	7.6	6.58	2000	66	69.00003726	237
CMC_MSSM_045	MSSM	Control	Male	Caucasian	1345	21.1	6.94	2005	73	68.00003672	192
CMC_MSSM_046	MSSM	Control	Female	Caucasian	876	1.8	6.49	2003	90	61.00003294	112
CMC_MSSM_047	MSSM	Control	Female	Caucasian	1199	14.9	6.82	2007	60	69.00003726	190
CMC_MSSM_049	MSSM	Control	Female	Caucasian	1222	20.3	NA	2007	61	64.00003456	114
CMC_MSSM_052	MSSM	Control	Female	Caucasian	1040	8	7.27	2006	85	60.0000324	109
CMC_MSSM_053	MSSM	Control	Male	Caucasian	1278	10.4	6.12	2006	64	73.00003942	251
CMC_MSSM_054	MSSM	Control	Male	Caucasian	1164	11.4	6.83	2005	84	69.00003726	135
CMC_MSSM_055	MSSM	Control	Male	Caucasian	1370	23.9	6.93	2005	66	72.00003888	167
CMC_MSSM_056	MSSM	Control	Female	Caucasian	1222	16.5	6.67	2005	67	65.0000351	283
CMC_MSSM_061	MSSM	Control	Male	Caucasian	1044	2.8	6.55	2005	68	64.00003456	112
CMC_MSSM_064	MSSM	Control	Male	Caucasian	1196	5.3	6.9	2003	85	67.00003618	158
CMC_MSSM_065	MSSM	Control	Female	Caucasian	984	5.9	6.58	2003	90	48.00002592	148
CMC_MSSM_066	MSSM	Control	Female	Caucasian	998	4.3	6.48	2003	78	62.00003348	126.6
CMC_MSSM_072	MSSM	Control	Female	Caucasian	1355	22.6	6.85	2004	66	68.00003672	NA
CMC_MSSM_073	MSSM	Control	Female	Caucasian	979	8.6	6.5	1988	79	NA	NA
CMC_MSSM_074	MSSM	Control	Female	Caucasian	1006	4.7	6.5	1988	86	NA	NA
CMC_MSSM_075	MSSM	Control	Female	Caucasian	1398	3.8	6.7	1991	77	NA	NA
CMC_MSSM_078	MSSM	Control	Male	Caucasian	1266	6.1	NA	2009	90	63.00003402	164.9
CMC_MSSM_079	MSSM	Control	Female	Caucasian	1235	5.8	NA	2010	90	62.00003348	162
CMC_MSSM_080	MSSM	Control	Male	Caucasian	1211	4.1	6.16	1997	84	NA	154
CMC_MSSM_081	MSSM	Control	Female	Caucasian	1022	5.1	6.4	1992	88	NA	NA
CMC_MSSM_084	MSSM	Control	Male	Caucasian	1388	19.5	6.59	2007	31	63.00003402	120
CMC_MSSM_085	MSSM	Control	Male	Caucasian	1238	19.2	6.14	2006	74	72.00003888	123
CMC_MSSM_089	MSSM	Control	Male	Caucasian	1104	3.5	NA	2008	90	71.00003834	150
CMC_MSSM_090	MSSM	Control	Male	Caucasian	1422	23.8	6.04	2006	70	NA	146.1
CMC_MSSM_092	MSSM	Control	Female	Caucasian	1049	6.8	6.6	2001	79	69.00003726	143
CMC_MSSM_093	MSSM	Control	Female	Caucasian	894	2	NA	2009	90	NA	173.8
CMC_MSSM_094	MSSM	Control	Female	Caucasian	1144	5	6.73	2005	90	63.00003402	135.2
CMC_MSSM_096	MSSM	Control	Female	Caucasian	1179	4.6	6.3	1988	67	NA	NA
CMC_MSSM_100	MSSM	Control	Male	Caucasian	1238	23.3	6.68	2006	64	70.0000378	96
CMC_MSSM_101	MSSM	Control	Female	Caucasian	1274	9	NA	2007	88	63.00003402	137
CMC_MSSM_105	MSSM	Control	Female	Caucasian	1128	5.1	NA	2010	90	64.00003456	160
CMC_MSSM_106	MSSM	Control	Female	Caucasian	1094	11.1	NA	2012	90	60.0000324	170.8
CMC_MSSM_107	MSSM	Control	Female	Caucasian	974	14.8	NA	2012	90	NA	NA
CMC_MSSM_108	MSSM	Control	Male	Caucasian	1120	11.8	NA	2012	90	70.0000378	140
CMC_MSSM_109	MSSM	Control	Female	Caucasian	1136	7.6	NA	2011	90	68.00003672	110
CMC_MSSM_110	MSSM	Control	Female	Caucasian	1210	4.8	6.4	1992	89	NA	NA
CMC_MSSM_117	MSSM	Control	Male	Caucasian	1170	6.6	NA	2008	86	67.00003618	116
CMC_MSSM_121	MSSM	Control	Male	Caucasian	1488	13.8	7	2006	51	71.00003834	190
CMC_MSSM_122	MSSM	Control	Male	Caucasian	1184	10.2	6.37	2006	90	NA	154
CMC_MSSM_124	MSSM	Control	Male	Caucasian	1386	16.8	6.55	2006	48	76.00004104	290
CMC_MSSM_130	MSSM	Control	Male	Caucasian	1464	10.7	6.78	2007	41	73.00003942	240
CMC_MSSM_136	MSSM	Control	Male	Caucasian	1164	2.9	6.32	2002	76	69.00003726	121.8
CMC_MSSM_137	MSSM	Control	Female	Caucasian	989	1.4	6.6	1993	90	NA	92
CMC_MSSM_140	MSSM	Control	Female	Caucasian	1128	5	NA	2009	85	63.00003402	163
CMC_MSSM_150	MSSM	Control	Female	Caucasian	1024	6.8	6.5	1988	90	NA	NA
CMC_MSSM_151	MSSM	Control	Male	Caucasian	1022	2.7	6	1989	89	NA	NA
CMC_MSSM_159	MSSM	Control	Female	Caucasian	1020	4.5	6.3	1999	90	NA	101
CMC_MSSM_160	MSSM	Control	Female	Caucasian	984	2.3	6.5	1991	80	NA	NA
CMC_MSSM_173	MSSM	Control	Male	Caucasian	1528	17.5	6.83	2006	53	71.00003834	200
CMC_MSSM_174	MSSM	Control	Male	Caucasian	1130	13	NA	2007	79	70.0000378	156
CMC_MSSM_175	MSSM	Control	Male	Caucasian	1286	8.7	6.6	1994	79	NA	NA

CMC_MSSM_177	MSSM	Control	Male	Caucasian	1448	20.2	NA	2007	60	67.00003618	195
CMC_MSSM_178	MSSM	Control	Male	Caucasian	1222	21	NA	2007	60	69.00003726	173
CMC_MSSM_179	MSSM	Control	Female	Caucasian	1004	11.5	NA	2008	86	63.00003402	154
CMC_MSSM_182	MSSM	Control	Male	Caucasian	1504	23.7	NA	2007	62	67.00003618	188
CMC_MSSM_183	MSSM	Control	Male	Caucasian	1168	10.8	NA	2007	66	71.00003834	130
CMC_MSSM_184	MSSM	Control	Female	Caucasian	1064	7.3	NA	2008	90	64.00003456	120
CMC_MSSM_185	MSSM	Control	Male	Caucasian	1247	11.7	6.5	1995	81	NA	133.2
CMC_MSSM_187	MSSM	Control	Male	Caucasian	1380	20.6	6.54	2005	39	71.00003834	175
CMC_MSSM_190	MSSM	Control	Male	Caucasian	1446	20.9	6.62	2005	84	71.00003834	175
CMC_MSSM_203	MSSM	Control	Male	Caucasian	1420	18	6.88	1996	70	NA	NA
CMC_MSSM_208	MSSM	Control	Female	Caucasian	809	2.7	6	1993	90	NA	84
CMC_MSSM_215	MSSM	Control	Male	Caucasian	941	9.6	6.5	1996	84	NA	100
CMC_MSSM_217	MSSM	Control	Female	Caucasian	1202	35.2	6.4	1995	79	NA	97
CMC_MSSM_219	MSSM	Control	Female	Caucasian	1289	20.4	6.2	1995	86	NA	98
CMC_MSSM_225	MSSM	Control	Female	Caucasian	1099	3.3	6.7	1991	90	NA	NA
CMC_MSSM_226	MSSM	Control	Female	Caucasian	962	3.8	6.6	1994	90	NA	74
CMC_MSSM_227	MSSM	Control	Male	Caucasian	1356	13	6.38	2005	27	67.00003618	172
CMC_MSSM_228	MSSM	Control	Male	Caucasian	1472	20	6.66	2005	43	71.00003834	190
CMC_MSSM_230	MSSM	Control	Male	Caucasian	1364	23.5	6.69	2004	38	71.00003834	170
CMC_MSSM_232	MSSM	Control	Male	Caucasian	1300	20	6.88	2004	24	NA	NA
CMC_MSSM_233	MSSM	Control	Male	Caucasian	1436	14.2	6.92	2004	49	68.00003672	229
CMC_MSSM_234	MSSM	Control	Male	Caucasian	1072	2.8	6.3	1994	90	NA	154
CMC_MSSM_235	MSSM	Control	Male	Caucasian	1450	22.5	6.26	2004	56	71.00003834	300
CMC_MSSM_237	MSSM	Control	Male	Caucasian	1272	18.8	6.56	2005	42	NA	NA
CMC_MSSM_238	MSSM	Control	Female	Caucasian	1094	9.5	6.21	2005	81	64.00003456	106.8
CMC_MSSM_242	MSSM	Control	Male	Caucasian	1410	21.7	7.09	2005	71	70.0000378	200
CMC_MSSM_244	MSSM	Control	Female	Caucasian	1286	19.3	6.33	2004	41	65.0000351	142
CMC_MSSM_247	MSSM	Control	Male	Caucasian	1368	28.8	6.76	2002	60	70.0000378	299
CMC_MSSM_249	MSSM	Control	Male	Caucasian	1135	2.9	6.65	1999	84	68.00003672	127
CMC_MSSM_251	MSSM	Control	Male	Caucasian	912	18.8	6.59	1989	61	NA	NA
CMC_MSSM_252	MSSM	Control	Female	Caucasian	1060	8.8	6.6	1991	76	NA	NA
CMC_MSSM_261	MSSM	Control	Female	Caucasian	1178	10.1	6.38	2005	79	63.00003402	115
CMC_MSSM_264	MSSM	Control	Male	Caucasian	1276	5	6.43	1993	75	71.00003834	93
CMC_MSSM_270	MSSM	Control	Female	Caucasian	1130	4.3	6.3	1996	85	65.0000351	113
CMC_MSSM_274	MSSM	Control	Female	Caucasian	1000	6.7	6.6	1991	89	NA	NA
CMC_MSSM_278	MSSM	Control	Female	Caucasian	1352	2.9	6.27	1995	60	NA	120
CMC_MSSM_280	MSSM	Control	Female	Caucasian	939	3.8	6.4	1995	85	NA	119
CMC_MSSM_281	MSSM	Control	Female	Caucasian	1026	7.1	6.5	1996	90	NA	90
CMC_MSSM_282	MSSM	Control	Male	Caucasian	1084	7.7	6.4	1995	88	NA	123
CMC_MSSM_292	MSSM	Control	Female	Caucasian	961	18.5	6.21	1998	84	62.00003348	180
CMC_MSSM_365	MSSM	Control	Female	Caucasian	1107	29	6.31	2000	82	NA	100
CMC_MSSM_369	MSSM	Control	Male	Caucasian	1100	4.2	6.6	2003	90	68.00003672	116.4
CMC_MSSM_376	MSSM	Control	Female	Caucasian	1080	3.4	6.3	1997	73	58.00003132	140
CMC_MSSM_383	MSSM	Control	Female	Caucasian	1188	6.2	6.76	2000	83	62.00003348	210
CMC_MSSM_384	MSSM	Control	Female	Caucasian	1078	3.5	6.2	1995	90	61.00003294	133
CMC_MSSM_389	MSSM	Control	Male	Caucasian	1500	18	6.74	1996	54	NA	NA
CMC_MSSM_391	MSSM	Control	Male	Caucasian	1404	33	6.27	2003	75	70.0000378	155
CMC_MSSM_401	MSSM	Control	Female	Caucasian	1014	5	6.2	1989	85	NA	NA
CMC_MSSM_402	MSSM	Control	Male	Caucasian	1344	20.4	6.61	2003	59	68.00003672	120
CMC_MSSM_407	MSSM	Control	Female	Caucasian	1030	7.8	NA	2009	90	59.00003186	103
CMC_MSSM_410	MSSM	Control	Male	Caucasian	1119	14.5	6.1	1993	86	NA	NA
CMC_MSSM_412	MSSM	Control	Female	Caucasian	1090	10	6.3	1988	73	NA	NA
CMC_MSSM_413	MSSM	Control	Male	Caucasian	1176	20	6.81	2003	90	68.00003672	143
CMC_MSSM_419	MSSM	Control	Female	Caucasian	1003	4.2	6	1991	90	NA	NA
CMC_PENN_001	Penn	Control	Female	Caucasian	1206	22	NA	2011	65	NA	NA
CMC_PENN_003	Penn	Control	Male	Caucasian	1545	15	NA	2011	67	72.83	220
CMC_PENN_004	Penn	Control	Female	Caucasian	1034	15	NA	2010	90	NA	NA
CMC_PENN_005	Penn	Control	Female	Caucasian	1201	19	NA	2011	78	63	200
CMC_PENN_006	Penn	Control	Female	Caucasian	1151	15	NA	2011	68	60.24	108
CMC_PENN_007	Penn	Control	Female	Caucasian	1207	19	5.97	2007	65	NA	NA
CMC_PENN_009	Penn	Control	Female	Caucasian	1026	3	NA	2010	83	NA	NA
CMC_PENN_011	Penn	Control	Male	Caucasian	1554	16	NA	2011	42	68.11	187
CMC_PENN_013	Penn	Control	Male	Caucasian	1330	21	NA	2010	68	NA	NA
CMC_PENN_015	Penn	Control	Male	Caucasian	1320	13.5	NA	2011	72	NA	NA
CMC_PENN_016	Penn	Control	Female	Caucasian	1300	5	NA	2010	82	NA	NA
CMC_PENN_017	Penn	Control	Female	Caucasian	1107	5	6.5	2002	90	NA	NA

CMC_PENN_018	Penn	Control	Male	Caucasian	1388	10.5	NA	2003	70	NA	NA
CMC_PENN_019	Penn	Control	Female	Caucasian	1198	14	NA	2006	85	NA	NA
CMC_PENN_021	Penn	Control	Male	Caucasian	1625	11	6.49	1998	69	NA	NA
CMC_PENN_022	Penn	Control	Female	Caucasian	1280	6	6.93	1992	36	72.44	285
CMC_PENN_025	Penn	Control	Male	Caucasian	1202	7	6.34	2003	85	NA	NA
CMC_PENN_026	Penn	Control	Male	Caucasian	NA	12	6.48	1986	29	65.75	187
CMC_PENN_028	Penn	Control	Male	Caucasian	1333	11	NA	2011	47	68.11	195
CMC_PENN_029	Penn	Control	Male	Caucasian	1369	6	NA	2010	61	NA	NA
CMC_PENN_077	Penn	Control	Male	Caucasian	1502	18	NA	2012	59	70.08	210
CMC_PENN_080	Penn	Control	Female	Caucasian	1245	24	NA	2011	67	57.87	129
CMC_PENN_081	Penn	Control	Male	Caucasian	1755	36	NA	2012	70	NA	NA
CMC_PENN_085	Penn	Control	Female	Caucasian	1250	6	6.16	1993	74	NA	NA
CMC_PENN_086	Penn	Control	Female	Caucasian	1080	11	6.45	1990	85	NA	NA
CMC_PENN_088	Penn	Control	Male	Caucasian	1488	19	6.68	1996	70	68.5	160
CMC_PITT_001	Pitt	Control	Male	Caucasian	1290	21.2	6.5	2004	49	63	165
CMC_PITT_002	Pitt	Control	Male	Caucasian	1500	23.5	6.7	2004	42	74	278
CMC_PITT_008	Pitt	Control	Male	Caucasian	1355	20	6.3	2002	59	70	180
CMC_PITT_009	Pitt	Control	Male	Caucasian	1410	13.8	6.6	2002	43	72	217
CMC_PITT_010	Pitt	Control	Male	Caucasian	1515	24.2	6.8	2003	51	70	178
CMC_PITT_013	Pitt	Control	Female	Caucasian	1240	9.1	6.5	2003	24	67	116
CMC_PITT_015	Pitt	Control	Male	Caucasian	1360	15.4	6.7	2003	55	68.5	195
CMC_PITT_022	Pitt	Control	Female	Caucasian	1220	16.4	6.2	2004	52	63	164
CMC_PITT_028	Pitt	Control	Female	Caucasian	1315	22.7	6.4	2004	58	70	250
CMC_PITT_032	Pitt	Control	Female	Caucasian	1245	19.7	6.7	2005	73	65	148
CMC_PITT_033	Pitt	Control	Female	Caucasian	1240	24.5	6.8	2005	39	64	178
CMC_PITT_034	Pitt	Control	Male	Caucasian	1330	6.4	6.8	2005	55	67	161
CMC_PITT_038	Pitt	Control	Male	Caucasian	1310	22.3	7	2005	43	69.5	212
CMC_PITT_039	Pitt	Control	Male	Caucasian	1515	16.4	6.7	2006	58	68	211
CMC_PITT_040	Pitt	Control	Male	Caucasian	1430	18.4	6.8	2006	65	72	205
CMC_PITT_042	Pitt	Control	Male	Caucasian	1470	24.2	6.4	2006	21	67	150
CMC_PITT_045	Pitt	Control	Male	Caucasian	1505	20.5	6.6	2006	37	73	289
CMC_PITT_046	Pitt	Control	Male	Caucasian	1385	21.7	6.6	2006	43	69	278
CMC_PITT_047	Pitt	Control	Male	Caucasian	1450	21.2	6.7	2006	46	70	200
CMC_PITT_048	Pitt	Control	Female	Caucasian	1335	7.8	6.6	2006	51	65	170
CMC_PITT_050	Pitt	Control	Male	Caucasian	1440	24.1	6.8	2007	65	71	216
CMC_PITT_055	Pitt	Control	Male	Caucasian	1495	23.8	6.5	2008	61	68	160
CMC_PITT_058	Pitt	Control	Male	Caucasian	1355	20.7	6.4	2008	50	70	175
CMC_PITT_059	Pitt	Control	Male	Caucasian	1555	9.4	6.4	2009	66	67	110
CMC_PITT_061	Pitt	Control	Female	Caucasian	1295	17.9	6.8	2009	45	61	122
CMC_PITT_062	Pitt	Control	Male	Caucasian	1460	23.2	6.5	2009	50	71.5	263
CMC_PITT_063	Pitt	Control	Male	Caucasian	1460	15.1	6.9	2009	17	78	178
CMC_PITT_064	Pitt	Control	Male	Caucasian	1470	24.4	6.9	2009	54	73	156
CMC_PITT_067	Pitt	Control	Male	Caucasian	1550	19.1	6.8	2009	58	66.5	146
CMC_PITT_068	Pitt	Control	Male	Caucasian	1450	25.3	6.8	2010	66	68	176
CMC_PITT_075	Pitt	Control	Female	Caucasian	1240	28.1	6.5	2012	36	69	142
CMC_PITT_080	Pitt	Control	Female	Caucasian	1190	22.5	6.7	1995	37	NA	NA
CMC_PITT_082	Pitt	Control	Male	Caucasian	1415	16.4	6.6	1995	61	NA	NA
CMC_PITT_088	Pitt	Control	Male	Caucasian	1560	18.8	7.1	1996	39	NA	NA
CMC_PITT_092	Pitt	Control	Male	Caucasian	1325	21.2	7	1996	65	NA	NA
CMC_PITT_093	Pitt	Control	Male	Caucasian	1620	16.2	7	1996	52	NA	NA
CMC_PITT_097	Pitt	Control	Male	Caucasian	1560	11.5	7.2	1997	51	70	320
CMC_PITT_098	Pitt	Control	Male	Caucasian	1285	14.5	6.6	1997	56	NA	186
CMC_PITT_099	Pitt	Control	Female	Caucasian	1240	22.6	7	1997	52	NA	196
CMC_PITT_100	Pitt	Control	Male	Caucasian	1565	20.7	7	1997	38	NA	NA
CMC_PITT_101	Pitt	Control	Male	Caucasian	1670	26.1	7	1997	42	NA	NA
CMC_PITT_104	Pitt	Control	Male	Caucasian	1450	15.8	6.9	1997	40	NA	NA
CMC_PITT_108	Pitt	Control	Male	Caucasian	1555	24	6.9	1999	57	NA	NA
CMC_PITT_109	Pitt	Control	Female	Caucasian	1285	24	7.1	1999	67	NA	NA
CMC_PITT_112	Pitt	Control	Male	Caucasian	1490	8.2	6.8	1999	54	NA	NA
CMC_PITT_113	Pitt	Control	Male	Caucasian	1575	16.6	6.7	2000	48	NA	NA
CMC_PITT_116	Pitt	Control	Male	Caucasian	1680	23.6	6.7	2000	60	69	171
CMC_PITT_121	Pitt	Control	Male	Caucasian	1500	25.9	6.4	2001	42	70	199
CMC_PITT_122	Pitt	Control	Female	Caucasian	1305	21.5	6.8	2001	65	62	145
CMC_PITT_123	Pitt	Control	Male	Caucasian	1440	22.5	6.2	2001	82	67	189
CMC_PITT_129	Pitt	Control	Male	Caucasian	1460	23.2	6.8	2002	53	69	236
CMC_PITT_133	Pitt	Control	Female	Caucasian	1435	14.9	6.8	2003	57	62	147

CMC_PITT_137	Pitt	Control	Male	Caucasian	1460	28	6.1	2003	55	71	245
CMC_PITT_140	Pitt	Control	Female	Caucasian	1280	14.5	6.4	2004	36	64	136
CMC_PITT_143	Pitt	Control	Female	Caucasian	1360	18.5	6.5	2005	65	63	111
CMC_PITT_144	Pitt	Control	Male	Caucasian	1325	24.5	6.5	2005	48	70	254
CMC_PITT_146	Pitt	Control	Male	Caucasian	1475	17.3	6.6	2006	45	65	140
CMC_PITT_147	Pitt	Control	Female	Caucasian	1345	12.3	6.7	2007	45	68	148
CMC_PITT_149	Pitt	Control	Male	Caucasian	1575	22	6.3	2007	46	71	218
CMC_PITT_153	Pitt	Control	Male	Caucasian	1350	20.2	6.6	2008	25	68	234
CMC_PITT_155	Pitt	Control	Male	Caucasian	1575	16.9	6.7	2008	25	68	123
CMC_PITT_161	Pitt	Control	Male	Caucasian	1430	23.8	6.9	2010	50	69	199
CMC_PITT_163	Pitt	Control	Female	Caucasian	1345	23.9	6.9	2010	21	65	161
CMC_PITT_165	Pitt	Control	Male	Caucasian	1460	16.6	6.9	2010	46	69	223
CMC_PITT_166	Pitt	Control	Female	Caucasian	1130	8.5	6.7	2011	67	66	157
CMC_PITT_168	Pitt	Control	Male	Caucasian	1600	28.2	6.8	2012	52	74	234
CMC_PITT_169	Pitt	Control	Female	Caucasian	1290	15.9	6.8	2012	23	70	206
CMC_PITT_170	Pitt	Control	Female	Caucasian	1340	13.7	6.8	2012	53	63	193
CMC_PITT_173	Pitt	Control	Male	Caucasian	1505	20.1	6.8	1998	22	77	197
CMC_PITT_174	Pitt	Control	Male	Caucasian	1400	11.8	6.8	1998	68	70	200
CMC_PITT_175	Pitt	Control	Male	Caucasian	1440	16.5	7	1999	58	70.5	173
CMC_PITT_176	Pitt	Control	Female	Caucasian	1400	15.4	6.6	1999	41	64	152
CMC_PITT_177	Pitt	Control	Male	Caucasian	1215	21.2	7.2	1999	70	68	175
CMC_MSSM_025	MSSM	SCZ	Male	Caucasian	1216	8.9	6.27	2002	68	NA	NA
CMC_MSSM_027	MSSM	SCZ	Male	Caucasian	1239	12.1	6.5	1994	66	NA	NA
CMC_MSSM_028	MSSM	SCZ	Female	Caucasian	1038	21.2	6.1	1995	76	NA	NA
CMC_MSSM_042	MSSM	SCZ	Male	Caucasian	1437	2.8	6.4	1993	61	NA	NA
CMC_MSSM_048	MSSM	SCZ	Male	Caucasian	1259	17.7	6.71	2001	84	NA	NA
CMC_MSSM_059	MSSM	SCZ	Male	Caucasian	1377	21.4	6.43	2000	57	NA	NA
CMC_MSSM_062	MSSM	SCZ	Male	Caucasian	1457	11	7.1	1994	75	NA	NA
CMC_MSSM_097	MSSM	SCZ	Male	Caucasian	1036	50.6	6.1	1992	73	NA	NA
CMC_MSSM_111	MSSM	SCZ	Male	Caucasian	1434	20.9	6.61	2006	54	74.00003996	207
CMC_MSSM_113	MSSM	SCZ	Male	Caucasian	1146	5.8	6.45	2002	75	65.0000351	106
CMC_MSSM_115	MSSM	SCZ	Female	Caucasian	1398	22.3	NA	2004	50	68.00003672	140
CMC_MSSM_116	MSSM	SCZ	Female	Caucasian	1226	16.6	NA	2008	74	62.00003348	179
CMC_MSSM_118	MSSM	SCZ	Male	Caucasian	1482	14.8	NA	2009	54	NA	NA
CMC_MSSM_119	MSSM	SCZ	Male	Caucasian	1081	48.3	6.9	1992	82	NA	NA
CMC_MSSM_132	MSSM	SCZ	Female	Caucasian	1076	15.1	6.47	2003	81	64.00003456	125.4
CMC_MSSM_133	MSSM	SCZ	Male	Caucasian	1324	14.3	6.49	2001	70	70.0000378	110.5
CMC_MSSM_139	MSSM	SCZ	Female	Caucasian	1055	14.4	6.7	1990	71	NA	NA
CMC_MSSM_142	MSSM	SCZ	Female	Caucasian	1050	3.4	6.2	1993	79	64.00003456	90
CMC_MSSM_161	MSSM	SCZ	Female	Caucasian	1091	33.3	6.6	1991	86	NA	NA
CMC_MSSM_163	MSSM	SCZ	Male	Caucasian	1207	33.8	6.7	1992	83	NA	NA
CMC_MSSM_166	MSSM	SCZ	Male	Caucasian	1339	50.8	6.5	1992	73	NA	NA
CMC_MSSM_168	MSSM	SCZ	Male	Caucasian	1001	3.3	6.1	1992	73	NA	NA
CMC_MSSM_169	MSSM	SCZ	Male	Caucasian	1186	49.2	6.6	1993	76	NA	NA
CMC_MSSM_180	MSSM	SCZ	Male	Caucasian	1416	40.4	7	1993	90	NA	NA
CMC_MSSM_192	MSSM	SCZ	Male	Caucasian	1216	7	6.3	1993	86	NA	NA
CMC_MSSM_193	MSSM	SCZ	Male	Caucasian	1146	9.8	NA	2010	70	68.00003672	143
CMC_MSSM_194	MSSM	SCZ	Female	Caucasian	1085	7.3	6.1	1990	90	60.0000324	95
CMC_MSSM_196	MSSM	SCZ	Female	Caucasian	920	5.5	6.2	1990	86	NA	NA
CMC_MSSM_197	MSSM	SCZ	Male	Caucasian	1374	20.6	6.6	1992	74	NA	NA
CMC_MSSM_198	MSSM	SCZ	Male	Caucasian	1408	53.6	7	1992	63	NA	NA
CMC_MSSM_199	MSSM	SCZ	Female	Caucasian	1021	49.9	6.2	1992	82	NA	NA
CMC_MSSM_200	MSSM	SCZ	Male	Caucasian	1021	49.9	6.4	1992	79	NA	NA
CMC_MSSM_201	MSSM	SCZ	Female	Caucasian	1258	44.4	6.4	1992	84	71.00003834	140
CMC_MSSM_204	MSSM	SCZ	Male	Caucasian	1290	50	6.5	1992	87	67.00003618	130
CMC_MSSM_205	MSSM	SCZ	Male	Caucasian	1299	34	6.6	1992	81	NA	NA
CMC_MSSM_209	MSSM	SCZ	Male	Caucasian	1081	3.4	6.4	1994	77	NA	NA
CMC_MSSM_210	MSSM	SCZ	Male	Caucasian	1304	33.3	6.1	1996	66	NA	83
CMC_MSSM_213	MSSM	SCZ	Male	Caucasian	1440	43	6.1	1996	62	NA	NA
CMC_MSSM_216	MSSM	SCZ	Female	Caucasian	1118	36.1	6.2	1994	64	NA	NA
CMC_MSSM_218	MSSM	SCZ	Male	Caucasian	1283	49.5	6.4	1995	76	NA	NA
CMC_MSSM_222	MSSM	SCZ	Male	Caucasian	1040	14.6	6.85	1997	68	NA	NA
CMC_MSSM_250	MSSM	SCZ	Male	Caucasian	1157	23.1	6.6	2000	41	NA	NA
CMC_MSSM_253	MSSM	SCZ	Female	Caucasian	1472	5.6	6.8	1992	75	62.00003348	127
CMC_MSSM_254	MSSM	SCZ	Male	Caucasian	1330	47.7	6.9	1991	64	NA	NA
CMC_MSSM_255	MSSM	SCZ	Female	Caucasian	1053	48.3	6	1990	77	62.00003348	100

CMC_MSSM_258	MSSM	SCZ	Female	Caucasian	1362	6.5	6.6	2005	64	63.00003402	198
CMC_MSSM_260	MSSM	SCZ	Female	Caucasian	960	21.5	6.49	2002	75	65.0000351	136
CMC_MSSM_262	MSSM	SCZ	Male	Caucasian	1942	26.1	6.64	2005	78	67.00003618	138
CMC_MSSM_265	MSSM	SCZ	Male	Caucasian	983	23.8	6.2	1992	84	NA	NA
CMC_MSSM_266	MSSM	SCZ	Male	Caucasian	1133	26	6.1	1992	81	NA	NA
CMC_MSSM_269	MSSM	SCZ	Male	Caucasian	1356	7.4	6.5	2006	79	NA	NA
CMC_MSSM_271	MSSM	SCZ	Female	Caucasian	1120	23.7	6.74	2006	62	65.0000351	176
CMC_MSSM_277	MSSM	SCZ	Male	Caucasian	1057	30	6.9	1997	58	NA	NA
CMC_MSSM_279	MSSM	SCZ	Male	Caucasian	939	11.8	6.2	1995	77	NA	NA
CMC_MSSM_288	MSSM	SCZ	Male	Caucasian	1062	31.8	6.31	2006	69	71.00003834	143.3
CMC_MSSM_289	MSSM	SCZ	Male	Caucasian	1202	28	6.58	2006	82	68.00003672	175
CMC_MSSM_291	MSSM	SCZ	Male	Caucasian	1576	32.3	6.48	2004	84	64.00003456	114
CMC_MSSM_294	MSSM	SCZ	Male	Caucasian	1420	30.5	6.51	2005	46	70.0000378	280
CMC_MSSM_295	MSSM	SCZ	Male	Caucasian	1166	34.1	6.8	2005	62	NA	142
CMC_MSSM_296	MSSM	SCZ	Female	Caucasian	1390	19.8	6.27	2005	88	61.00003294	129
CMC_MSSM_297	MSSM	SCZ	Male	Caucasian	1144	27	6.75	2010	47	73.00003942	270
CMC_MSSM_298	MSSM	SCZ	Female	Caucasian	1084	40.8	NA	2007	65	63.00003402	129
CMC_MSSM_299	MSSM	SCZ	Female	Caucasian	1440	29.9	6.4	2011	59	62.00003348	168
CMC_MSSM_300	MSSM	SCZ	Male	Caucasian	932	43.6	NA	2007	72	NA	NA
CMC_MSSM_304	MSSM	SCZ	Male	Caucasian	1114	47.3	NA	2009	76	73.00003942	216
CMC_MSSM_305	MSSM	SCZ	Female	Caucasian	1382	7.6	NA	2010	86	64.00003456	102
CMC_MSSM_306	MSSM	SCZ	Male	Caucasian	1422	25.5	NA	2010	65	71.00003834	208
CMC_MSSM_309	MSSM	SCZ	Female	Caucasian	1084	3.7	NA	2003	84	65.0000351	128
CMC_MSSM_310	MSSM	SCZ	Female	Caucasian	1196	48.8	6.49	2003	90	NA	NA
CMC_MSSM_311	MSSM	SCZ	Female	Caucasian	1492	25.8	6.78	2002	62	63.00003402	177
CMC_MSSM_312	MSSM	SCZ	Male	Caucasian	1380	48.1	6.72	2002	68	71.00003834	197
CMC_MSSM_313	MSSM	SCZ	Male	Caucasian	1112	48.3	6.41	2002	67	72.00003888	185
CMC_MSSM_319	MSSM	SCZ	Male	Caucasian	810	27	6.19	2003	52	74.00003996	273
CMC_MSSM_321	MSSM	SCZ	Male	Caucasian	1260	25.5	6.53	2002	48	73.00003942	224
CMC_MSSM_322	MSSM	SCZ	Male	Caucasian	1042	15.2	6.49	2001	85	NA	NA
CMC_MSSM_323	MSSM	SCZ	Male	Caucasian	1054	7.7	6.2	2001	89	NA	NA
CMC_MSSM_326	MSSM	SCZ	Male	Caucasian	1044	48.6	6.13	2001	82	NA	NA
CMC_MSSM_331	MSSM	SCZ	Female	Caucasian	1180	29.9	6.53	1996	67	NA	111
CMC_MSSM_332	MSSM	SCZ	Female	Caucasian	1287	46.4	6.8	2000	73	NA	NA
CMC_MSSM_335	MSSM	SCZ	Male	Caucasian	1054	27.3	6.65	2000	32	65.0000351	282
CMC_MSSM_336	MSSM	SCZ	Female	Caucasian	1125	9.6	6.2	2000	89	NA	NA
CMC_MSSM_337	MSSM	SCZ	Female	Caucasian	1355	10.3	6.11	1998	59	NA	NA
CMC_MSSM_339	MSSM	SCZ	Male	Caucasian	1325	31.8	NA	1999	58	NA	NA
CMC_MSSM_343	MSSM	SCZ	Female	Caucasian	1491	21.9	6.34	2000	59	NA	NA
CMC_MSSM_344	MSSM	SCZ	Male	Caucasian	1222	48.6	6.48	2000	90	56.00003024	116
CMC_MSSM_345	MSSM	SCZ	Male	Caucasian	1338	9.2	6.09	2000	79	NA	NA
CMC_MSSM_346	MSSM	SCZ	Male	Caucasian	1076	3.5	6.35	2000	68	NA	NA
CMC_MSSM_347	MSSM	SCZ	Male	Caucasian	1304	11.4	6.74	2001	82	NA	NA
CMC_MSSM_348	MSSM	SCZ	Female	Caucasian	1254	45	6.45	1996	89	NA	NA
CMC_MSSM_349	MSSM	SCZ	Male	Caucasian	1069	46.7	6.2	1993	79	66.00003564	138
CMC_MSSM_352	MSSM	SCZ	Male	Caucasian	1014	4.3	6.29	1997	64	NA	NA
CMC_MSSM_354	MSSM	SCZ	Male	Caucasian	1166	25.5	6.8	1992	83	NA	NA
CMC_MSSM_355	MSSM	SCZ	Female	Caucasian	1108	25.8	6.5	2008	90	64.00003456	90
CMC_MSSM_356	MSSM	SCZ	Female	Caucasian	1139	42.3	NA	1992	81	NA	86
CMC_MSSM_359	MSSM	SCZ	Male	Caucasian	1282	8.8	6.15	2001	73	NA	NA
CMC_MSSM_360	MSSM	SCZ	Female	Caucasian	1008	7.8	6.57	2001	90	NA	NA
CMC_MSSM_361	MSSM	SCZ	Male	Caucasian	1262	24	6.4	2001	77	NA	NA
CMC_MSSM_362	MSSM	SCZ	Male	Caucasian	1194	7.2	6.35	2001	73	NA	NA
CMC_MSSM_363	MSSM	SCZ	Female	Caucasian	1068	9.7	6.01	2001	77	NA	NA
CMC_MSSM_364	MSSM	SCZ	Male	Caucasian	1650	13.5	6.45	2002	56	NA	NA
CMC_MSSM_366	MSSM	SCZ	Male	Caucasian	1144	5.3	7	1991	84	NA	NA
CMC_MSSM_367	MSSM	SCZ	Male	Caucasian	1221	5.3	6.2	1994	86	NA	162
CMC_MSSM_370	MSSM	SCZ	Female	Caucasian	1204	5.5	6.6	1992	71	NA	NA
CMC_MSSM_372	MSSM	SCZ	Male	Caucasian	1020	17.7	6.59	1998	90	NA	NA
CMC_MSSM_373	MSSM	SCZ	Male	Caucasian	1070	26	6.67	1999	90	NA	NA
CMC_MSSM_377	MSSM	SCZ	Male	Caucasian	1134	8.4	6.69	1998	66	NA	NA
CMC_MSSM_379	MSSM	SCZ	Male	Caucasian	1222	9.5	NA	2007	71	67.00003618	156
CMC_MSSM_382	MSSM	SCZ	Male	Caucasian	1309	7.9	6.5	1994	73	NA	NA
CMC_MSSM_385	MSSM	SCZ	Male	Caucasian	1042	16.6	6.7	2000	76	NA	NA
CMC_MSSM_386	MSSM	SCZ	Male	Caucasian	1261	5.3	6.3	1994	85	NA	NA
CMC_MSSM_388	MSSM	SCZ	Female	Caucasian	1210	6.9	6.4	1989	80	49.00002646	184

CMC_MSSM_390	MSSM	SCZ	Male	Caucasian	1401	14.1	6.65	1997	86	NA	127
CMC_MSSM_403	MSSM	SCZ	Male	Caucasian	1303	40.2	6.68	2000	69	NA	NA
CMC_MSSM_404	MSSM	SCZ	Male	Caucasian	1109	3	6.1	1991	61	NA	NA
CMC_MSSM_415	MSSM	SCZ	Male	Caucasian	1238	6.7	6.2	1990	58	NA	NA
CMC_MSSM_416	MSSM	SCZ	Female	Caucasian	1033	8.5	6.1	1991	76	NA	NA
CMC_MSSM_420	MSSM	SCZ	Male	Caucasian	1223	6.2	6.5	1992	63	NA	NA
CMC_MSSM_421	MSSM	SCZ	Female	Caucasian	966	13.7	6.2	1992	69	NA	NA
CMC_MSSM_422	MSSM	SCZ	Male	Caucasian	1022	11.2	6.5	1992	87	NA	NA
CMC_PENN_030	Penn	SCZ	Female	Caucasian	1244	16	6.48	1997	88	NA	NA
CMC_PENN_031	Penn	SCZ	Female	Caucasian	1400	16	6.29	1997	80	NA	NA
CMC_PENN_032	Penn	SCZ	Male	Caucasian	1420	30	6.49	1990	86	65.35	125
CMC_PENN_034	Penn	SCZ	Male	Caucasian	1270	12	6.24	1992	71	NA	NA
CMC_PENN_035	Penn	SCZ	Female	Caucasian	1021	33.5	NA	2006	87	NA	NA
CMC_PENN_036	Penn	SCZ	Female	Caucasian	1002	10	6.77	1998	76	NA	NA
CMC_PENN_037	Penn	SCZ	Female	Caucasian	1060	11	6.5	1994	80	NA	NA
CMC_PENN_038	Penn	SCZ	Male	Caucasian	1341	15	6.42	1994	89	NA	NA
CMC_PENN_039	Penn	SCZ	Male	Caucasian	1386	9	NA	2004	81	NA	NA
CMC_PENN_040	Penn	SCZ	Female	Caucasian	1097	11	6.63	1997	75	NA	NA
CMC_PENN_041	Penn	SCZ	Male	Caucasian	1310	12	6.67	1998	86	NA	NA
CMC_PENN_042	Penn	SCZ	Female	Caucasian	990	7	6.88	1999	88	NA	NA
CMC_PENN_044	Penn	SCZ	Female	Caucasian	1219	16	6.55	1996	75	NA	NA
CMC_PENN_045	Penn	SCZ	Female	Caucasian	1040	8	6.25	1991	79	61.02	175
CMC_PENN_046	Penn	SCZ	Female	Caucasian	1120	17.5	6.26	2001	70	NA	NA
CMC_PENN_047	Penn	SCZ	Male	Caucasian	1360	19	6.32	1990	78	NA	75
CMC_PENN_048	Penn	SCZ	Female	Caucasian	951	6	NA	2008	66	NA	NA
CMC_PENN_050	Penn	SCZ	Male	Caucasian	1503	13.5	6.26	1996	69	NA	NA
CMC_PENN_051	Penn	SCZ	Female	Caucasian	935	9.5	6.61	1996	77	NA	NA
CMC_PENN_053	Penn	SCZ	Female	Caucasian	980	7.5	6.58	2001	86	NA	NA
CMC_PENN_054	Penn	SCZ	Male	Caucasian	1466	48	NA	2009	26	NA	NA
CMC_PENN_056	Penn	SCZ	Female	Caucasian	1140	15	6.62	1992	74	NA	NA
CMC_PENN_057	Penn	SCZ	Male	Caucasian	1385	5	6.6	2002	81	NA	NA
CMC_PENN_058	Penn	SCZ	Female	Caucasian	1008	20	NA	2008	89	NA	NA
CMC_PENN_059	Penn	SCZ	Female	Caucasian	1222	7.5	6.57	2002	83	NA	NA
CMC_PENN_060	Penn	SCZ	Female	Caucasian	1110	9.5	6.52	2002	76	NA	NA
CMC_PENN_061	Penn	SCZ	Male	Caucasian	1366	32	NA	2011	86	NA	NA
CMC_PENN_062	Penn	SCZ	Male	Caucasian	1080	19.5	6.54	1988	82	61.81	140
CMC_PENN_063	Penn	SCZ	Male	Caucasian	1458	18	NA	2008	83	NA	NA
CMC_PENN_064	Penn	SCZ	Female	Caucasian	1200	11	6.22	1990	76	61.81	140
CMC_PENN_065	Penn	SCZ	Female	Caucasian	1040	6.5	6.23	1993	74	NA	NA
CMC_PENN_066	Penn	SCZ	Female	Caucasian	1200	15	5.83	1993	75	NA	NA
CMC_PENN_067	Penn	SCZ	Female	Caucasian	1018	20	6.73	1994	78	NA	NA
CMC_PENN_068	Penn	SCZ	Male	Caucasian	1300	7	6.07	1993	79	NA	NA
CMC_PENN_070	Penn	SCZ	Female	Caucasian	1100	6	NA	2005	90	60	163.1
CMC_PENN_071	Penn	SCZ	Female	Caucasian	1270	6.5	6.7	1993	87	NA	NA
CMC_PENN_072	Penn	SCZ	Female	Caucasian	1200	7.5	6.58	1994	88	NA	NA
CMC_PENN_073	Penn	SCZ	Female	Caucasian	1100	12	6.19	1994	86	NA	NA
CMC_PENN_074	Penn	SCZ	Female	Caucasian	1020	11.5	6.7	1992	72	NA	NA
CMC_PENN_075	Penn	SCZ	Male	Caucasian	1382	7	6.63	1995	82	NA	NA
CMC_PENN_082	Penn	SCZ	Male	Caucasian	1338	17	NA	2007	77	NA	NA
CMC_PENN_089	Penn	SCZ	Male	Caucasian	1100	20	6.55	2005	81	NA	NA
CMC_PENN_090	Penn	SCZ	Female	Caucasian	1325	13	6.47	1994	81	NA	NA
CMC_PENN_091	Penn	SCZ	Female	Caucasian	976	16	5.87	1995	90	NA	NA
CMC_PENN_092	Penn	SCZ	Male	Caucasian	1240	10.5	6.28	1993	90	NA	NA
CMC_PENN_093	Penn	SCZ	Male	Caucasian	1311	12	6.81	1996	75	NA	NA
CMC_PENN_094	Penn	SCZ	Female	Caucasian	1086	11	6.92	1996	86	NA	NA
CMC_PENN_095	Penn	SCZ	Female	Caucasian	978	12.5	NA	2003	89	59.45	140
CMC_PENN_096	Penn	SCZ	Male	Caucasian	1188	10	6.64	1994	71	NA	NA
CMC_PENN_097	Penn	SCZ	Male	Caucasian	1200	16.5	6.13	1993	70	NA	NA
CMC_PENN_098	Penn	SCZ	Male	Caucasian	1080	8.5	6.77	1994	90	NA	NA
CMC_PENN_099	Penn	SCZ	Male	Caucasian	1200	7	6.61	1992	83	67.72	85
CMC_PENN_100	Penn	SCZ	Female	Caucasian	1100	7	6.32	1993	82	NA	NA
CMC_PENN_101	Penn	SCZ	Male	Caucasian	1260	14	6.66	1993	74	NA	NA
CMC_PITT_003	Pitt	SCZ	Male	Caucasian	1500	28.8	6.6	2004	38	68	130
CMC_PITT_007	Pitt	SCZ	Female	Caucasian	1300	23.8	6.6	2004	46	62.5	115.5
CMC_PITT_011	Pitt	SCZ	Male	Caucasian	1530	21.5	6.4	2003	49	71	194
CMC_PITT_014	Pitt	SCZ	Male	Caucasian	1220	7.9	6.2	2003	53	66	188

CMC_PITT_017	Pitt	SCZ	Male	Caucasian	1315	22.9	6.4	2003	62	71	164
CMC_PITT_019	Pitt	SCZ	Male	Caucasian	1575	7.7	6.2	2004	58	69	117
CMC_PITT_020	Pitt	SCZ	Female	Caucasian	1285	14.4	6.4	2004	47	66	234
CMC_PITT_023	Pitt	SCZ	Male	Caucasian	1695	9.1	6.5	2004	35	71	202
CMC_PITT_024	Pitt	SCZ	Female	Caucasian	1365	20.1	6.3	2004	41	64	189
CMC_PITT_025	Pitt	SCZ	Male	Caucasian	1400	30.8	6.4	2004	32	71.5	231
CMC_PITT_026	Pitt	SCZ	Male	Caucasian	1550	16.9	6.6	2004	50	64	186
CMC_PITT_029	Pitt	SCZ	Male	Caucasian	1560	27.4	6.4	2004	34	73	168
CMC_PITT_030	Pitt	SCZ	Male	Caucasian	1315	22.7	7.1	2005	62	67	114
CMC_PITT_037	Pitt	SCZ	Male	Caucasian	1215	11	6.2	2005	50	69	150
CMC_PITT_041	Pitt	SCZ	Female	Caucasian	1175	24.5	6.6	2006	44	65	115
CMC_PITT_043	Pitt	SCZ	Male	Caucasian	1335	23.2	6.4	2006	63	69	181
CMC_PITT_044	Pitt	SCZ	Male	Caucasian	1465	28.9	6.6	2006	47	69	232
CMC_PITT_049	Pitt	SCZ	Male	Caucasian	1535	23.4	6.7	2007	47	70	225
CMC_PITT_051	Pitt	SCZ	Male	Caucasian	1500	11.1	6.4	2007	62	72	172
CMC_PITT_052	Pitt	SCZ	Male	Caucasian	1600	24.1	6.1	2007	59	68	213
CMC_PITT_053	Pitt	SCZ	Male	Caucasian	1550	8.2	6.2	2007	42	68	185
CMC_PITT_056	Pitt	SCZ	Male	Caucasian	1655	39.9	6.7	2008	37	72	236
CMC_PITT_057	Pitt	SCZ	Female	Caucasian	1515	14.1	6.6	2008	47	63	275
CMC_PITT_060	Pitt	SCZ	Male	Caucasian	1550	17.4	6.7	2009	65	72	220
CMC_PITT_065	Pitt	SCZ	Female	Caucasian	1315	16.1	6.7	2009	69	60.5	119
CMC_PITT_066	Pitt	SCZ	Male	Caucasian	1320	18.4	6.8	2009	32	73	240
CMC_PITT_071	Pitt	SCZ	Male	Caucasian	1310	31.9	6.6	2011	51	72	254
CMC_PITT_073	Pitt	SCZ	Male	Caucasian	1370	15.1	6.2	2011	63	73	251
CMC_PITT_074	Pitt	SCZ	Male	Caucasian	1340	28.6	6.1	2012	54	77	192
CMC_PITT_077	Pitt	SCZ	Male	Caucasian	1480	28.6	6.8	1995	40	NA	NA
CMC_PITT_079	Pitt	SCZ	Male	Caucasian	1645	40.5	7.1	1995	50	NA	NA
CMC_PITT_083	Pitt	SCZ	Male	Caucasian	1420	18.3	6.8	1995	63	69	109
CMC_PITT_085	Pitt	SCZ	Male	Caucasian	1390	30.2	7.2	1996	46	65	158
CMC_PITT_089	Pitt	SCZ	Male	Caucasian	1315	16	7.3	1996	83	66	140
CMC_PITT_090	Pitt	SCZ	Male	Caucasian	1505	18.9	6.8	1996	58	69	234
CMC_PITT_094	Pitt	SCZ	Male	Caucasian	1260	4.5	6.9	1996	49	74	240
CMC_PITT_107	Pitt	SCZ	Female	Caucasian	1315	29	6.4	1999	63	67	161
CMC_PITT_111	Pitt	SCZ	Male	Caucasian	1160	5	6.8	1999	25	66	150
CMC_PITT_117	Pitt	SCZ	Male	Caucasian	1565	28	6.2	2000	33	71	275
CMC_PITT_118	Pitt	SCZ	Female	Caucasian	1150	23.8	6.8	2000	71	65	153
CMC_PITT_120	Pitt	SCZ	Male	Caucasian	1495	8.3	5.9	2001	44	72	336
